# MYC-driven synthesis of Siglec ligands is a glycoimmune checkpoint

**DOI:** 10.1073/pnas.2215376120

**Published:** 2023-03-10

**Authors:** Benjamin A. H. Smith, Anja Deutzmann, Kristina M. Correa, Corleone S. Delaveris, Renumathy Dhanasekaran, Christopher G. Dove, Delaney K. Sullivan, Simon Wisnovsky, Jessica C. Stark, John V. Pluvinage, Srividya Swaminathan, Nicholas M. Riley, Anand Rajan, Ravindra Majeti, Dean W. Felsher, Carolyn R. Bertozzi

**Affiliations:** ^a^Sarafan ChEM-H, Stanford University, Stanford, CA 94305; ^b^Department of Chemical and Systems Biology, Stanford University, Stanford, CA 94305; ^c^Division of Oncology, Department of Medicine, Stanford University School of Medicine, Stanford, CA 94305; ^d^Department of Chemistry, Stanford University, Stanford, CA 94305; ^e^Division of Gastroenterology and Hepatology, Department of Medicine, Stanford University School of Medicine, Stanford, CA 94305; ^f^Division of Hematology, Department of Medicine, Stanford University, Stanford, CA 94305; ^g^Institute for Stem Cell Biology and Regenerative Medicine, Stanford University, Stanford, CA 94305; ^h^Faculty of Pharmaceutical Sciences, University of British Columbia, British Columbia, BC V6T 1Z3, Canada; ^i^Department of Neurology, University of California, San Francisco, CA 94143; ^j^Department of Systems Biology, Beckman Research Institute of City of Hope, Monrovia, CA 91016; ^k^Department of Pediatrics, Beckman Research Institute of City of Hope, Duarte, CA 91010; ^l^Department of Pathology, University of Iowa, Iowa City, IA 52242; ^m^Department of Pathology, Stanford University School of Medicine, Stanford, CA 94305; ^n^Howard Hughes Medical Institute, Stanford University, Stanford, CA 94305

**Keywords:** glycosylation, MYC, oncogene, Siglec

## Abstract

The Siglecs (sialic acid-binding immunoglobulin-like lectins) are glycoimmune checkpoint receptors that suppress immune cell activation upon engagement of cognate sialoglycan ligands. The cellular drivers underlying Siglec ligand production on cancer cells are poorly understood. We find the *MYC* oncogene causally regulates Siglec ligand production to enable tumor immune evasion. A combination of glycomics and RNA-sequencing of mouse tumors revealed the *MYC* oncogene controls expression of the sialyltransferase *St6galnac4* and induces a glycan known as disialyl-T. Using in vivo models and primary human leukemias, we find that disialyl-T functions as a “don’t eat me” signal by engaging macrophage Siglec-E in mice or the human ortholog Siglec-7, thereby preventing cancer cell clearance. Combined high expression of *MYC* and *ST6GALNAC4* identifies patients with high-risk cancers and reduced tumor myeloid infiltration. MYC therefore regulates glycosylation to enable tumor immune evasion. We conclude that disialyl-T is a glycoimmune checkpoint ligand. Thus, disialyl-T is a candidate for antibody-based checkpoint blockade, and the disialyl-T synthase ST6GALNAC4 is a potential enzyme target for small molecule-mediated immune therapy.

## Significance

*MYC* is one of the most frequently dysregulated oncogenes in human cancer. We discover that MYC causally regulates glycosylation on the surface of cancer cells, which in turn facilitates immune evasion. Using a conditional transgenic model of MYC-induced tumorigenesis, we find that MYC drives the display of a particular glycan known as disialyl-T on tumor cells. Remarkably, the disialyl-T glycan engages specific Siglec receptors on myeloid cells to inhibit the anticancer immune response, thereby promoting tumor growth in vivo. These data identify a signature of malignant glycosylation on MYC-driven cancers that suggests potential targets for immunotherapy.

The Siglecs are a family of immune modulatory receptors that populate all classes of immune cells (1–4). Of the 14 Siglec family members in humans, nine possess intracellular immunoreceptor tyrosine-based inhibitory motifs (ITIMs) that signal to suppress immune cell activation, analogous to the activity of PD-1 and SIRPα on T cells and macrophages, respectively (3). Reports that Siglecs on macrophages (5–7), NK cells (8–11), and T cells (12, 13) contribute to cancer immune evasion have stimulated interest in targeting Siglecs and their ligands for immune therapy (14).

The tumor-associated sialoglycans that engage Siglec receptors are not well-understood nor are the mechanisms that drive their upregulation in the tumor microenvironment. There is a long history (>60 y) of studies observing increased abundance of sialic acids on cancer cells (15). Likewise, immunohistochemical studies with Siglec-Fc reagents have shown the upregulation of functional Siglec ligands in various cancers (9). However, the molecular structures of glycans that support Siglec binding in tumor microenvironments are largely unknown. Tumor-associated glycan signatures are products of biosynthetic machineries that are presumably altered in cancer. Changes in glycogene expression are widely observed in cancer (16, 17), but a cohesive picture that relates these transcriptomic changes to altered glycosylation of functional relevance is lacking. Furthermore, the mechanisms driving altered glycogene expression have not been addressed. Understanding the biological axis connecting oncogenesis, glycan biosynthesis, and Siglec-mediated immune evasion could reveal new targets for cancer immunotherapy.

We hypothesized that the *MYC* oncogene regulates glycosylation and, in particular, the biosynthesis of sialoglycans. MYC is a transcription factor that is dysregulated in over 70% of human cancers where its overexpression is associated with poor patient outcomes (18–20). MYC is a common oncogenic driver even in tumors where it is not genomically dysregulated or directly overexpressed (21). For example, the epistatic activation of MYC, for instance, via NOTCH1 signaling in T-cell acute lymphoblastic leukemia (T-ALL) is essential to tumorigenesis (22–24). By regulating programs of gene transcription, MYC contributes to various hallmarks of cancer, including cellular proliferation and growth, self-renewal and stemness, cellular metabolism and protein biogenesis, evasion of apoptosis, and genomic instability (25–35). Two earlier studies using fibroblast and colon adenocarcinoma lines found that MYC promotes N-glycan branching (36) and display of the glycan sialyl-Lewis^x^ (37), respectively, although the physiological relevance of these changes was unclear. We recently demonstrated that MYC also contributes to cancer’s escape from immune surveillance by regulating the expression of checkpoint protein ligands (38–40). Therefore, we wondered whether MYC’s program for immune suppression might extend to the synthesis of sialoglycans that engage Siglecs. Here, using a conditional transgenic mouse model of MYC-induced T-ALL that expresses human *MYC* in a tetracycline-dependent manner (EμSRα-*tTA*/tet-O-*MYC*) (41), we asked whether MYC directs altered glycosylation and how this affects the innate antitumor immune response and tumorigenesis (*SI Appendix*, Fig. S1*A*).

## Results

### MYC Drives *St6galnac4* Expression and Promotes Cell Surface Display of the Disialyl-T Glycan Structure.

**Fig. 1.**
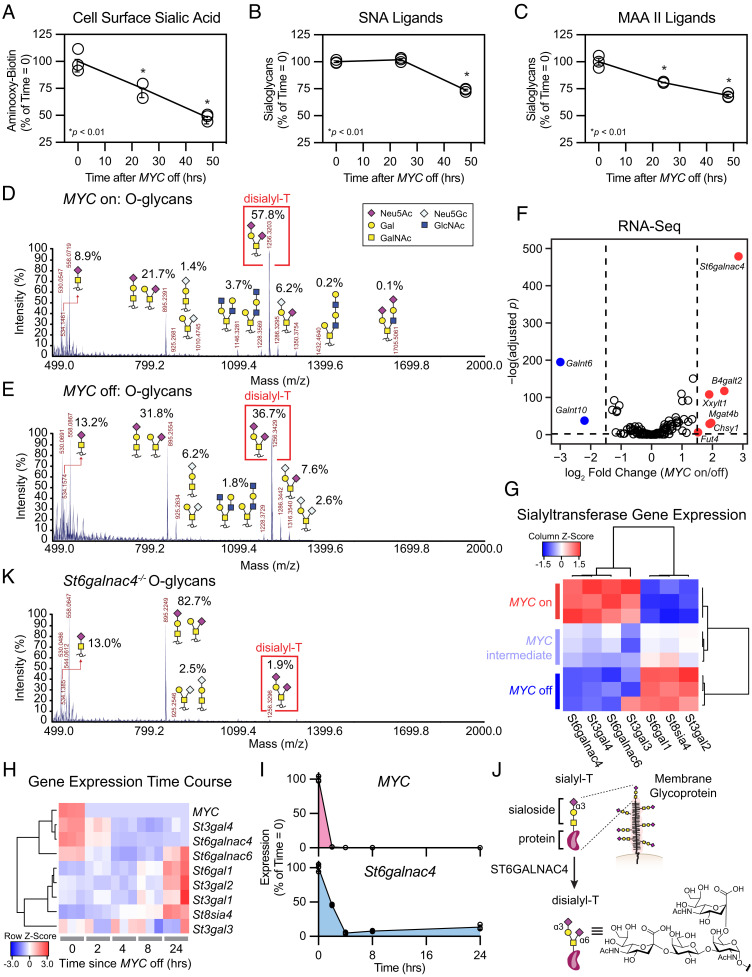
MYC regulated *St6galnac4* promotes display of the glycan disialyl-T. (*A*) Murine T-ALL cell surface sialic acids were quantified at various time points after doxycycline administration to turn off expression of the *MYC* transgene. Sialic acids were detected via oxidation with mild periodate treatment and subsequent labeling with an aminooxy-biotin probe (n = 3 per time point, two-tailed Student’s *t* test comparing each time point to t = 0 h, *P < 0.05, **P < 0.01). Data are normalized to control, mean ± SEM. (*B* and *C*) Sialoglycans were measured at various time points using the lectins SNA and MAA II after turning *MYC* off (n = 3 per time point, two-tailed Student’s *t* test comparing each time point to t = 0 h, *P < 0.05, **P < 0.01). Data are normalized to control, mean ± SD. (*D* and *E*) The O-glycome of *MYC* on (*D*) and off (*E*) cells was profiled by MALDI-TOF mass spectrometry following β-elimination. The relative abundance of recognizable glycan species within the spectrum is indicated. (*F*) RNA-sequencing of cells in the *MYC* on and off states. Volcano plot shows differential expression of annotated glycogenes. Genes exhibiting log_2_ fold changes greater than 1.5 and meeting a significance threshold of *P *< 0.01 at an FDR = 0.01 are highlighted (n = 3 per treatment group). (*G*) Heatmap of sialyltransferases meeting an expression threshold >2 transcripts per million (TPM). Data are displayed as a Z-score from column-normalized TPMs. (*H*) Heatmap of *MYC* transgene and relevant sialyltransferase expression generated by RNA-seq of T-ALL at the indicated time after doxycycline administration to turn *MYC* off. Data are displayed as a Z-score from row normalized TPMs (n = 3 per group). (*I*) Expression of the *MYC* transgene and *St6galnac4* were profiled by RNA-seq in T-ALL at the indicated time after turning off *MYC*. Data are normalized to t = 0 h, mean ± SD (n = 3 per group). (*J*) Schematic for elaboration of sialyl-T into disialyl-T by ST6GALNAC4. (*K*) The O-glycome of *St6galnac4^−/−^* cells was profiled by MALDI-TOF mass spectrometry following β-elimination.

To assess whether MYC expression affects the abundance of sialoglycans on cancer cells, we used a periodate-aminooxy ligation (42) to quantify sialic acids on the surface of MYC-driven T-ALL cells in the *MYC* on (no doxycycline) and off states (plus doxycycline) (*SI Appendix*, Fig. S1*B*). After 48 h, *MYC* off cells lost over 50% of surface-bound sialic acids relative to cells in the *MYC* on state (Fig. 1*A*). This was not caused by an overall reduction in metabolic activity in the *MYC* off cells, as *MYC* on and off cells had similar quantities of total sialic acid per cell as measured in total cell lysates (*SI Appendix*, Fig. S2 *A*–*C*). Instead, high MYC activity appeared to specifically promote the display of sialoglycans on the cell surface.

Subsets of sialoglycans are recognized by the lectins MAA II (glycans containing Neu5Acα2–3Galβ1–3GalNAc) (43) and SNA (glycans containing Neu5Acα2–6Gal) (44), that we used to further probe the effects of *MYC* expression on cell surface sialoside display. We observed a decrease in MAA II staining by flow cytometry within 24 h after turning *MYC* off and a decrease in SNA staining that was delayed until 48 h (Fig. 1 *B* and *C*). Also, we assayed the recovery of MAA II binding to *MYC* on and off cells following treatment with a sialidase to remove all cell surface sialic acids. While MAA II binding to *MYC* on cells recovered to similar levels as untreated controls within 30 h, MAA II binding to *MYC* off cells only recovered partially within this time period (*SI Appendix*, Fig. S3 *A* and *B*). MYC therefore activates biosynthetic processes that enhance the expression of MAA II-binding sialoglycans on the tumor cell surface.

To identify the specific sialoglycan structures that are regulated by MYC overexpression, we performed glycomics analyses by mass spectrometry focusing on O-glycans (linked to Ser/Thr) and N-glycans (linked to Asn). O- and N-glycans were released from glycoproteins in whole-cell lysates by β-elimination and PNGase digestion, respectively. The glycans were methylated and then identified and quantitated by MALDI-TOF mass spectrometry. The structures of the glycans we detected were confirmed using NSI-FTMS/MS (*SI Appendix*, Fig. S4 *A* and *B*).

We observed a striking difference in O-glycan profile between the *MYC* on and off states. In *MYC* on cells, the O-glycans were dominated by a sialoglycan named disialyl-T (Neu5Acα2–3Galβ1–3[Neu5Acα2–6]GalNAc), which represented approximately 60% of the total signal present (Fig. 1*D*). Conversely, in *MYC* off cells, disialyl-T represented only 37% of the total O-glycans (Fig. 1*E*). We confirmed these changes by assaying the recovery of disialyl-T on *MYC* on and off cells following sialidase treatment. At 48 h after sialidase treatment, disialyl-T represented 23% of the O-glycans on *MYC* on cells compared to 5% of the O-glycans on *MYC* off cells (*SI Appendix*, Fig. S5 *A*–*C*).

By contrast, N-glycan profiling revealed only subtle differences between the *MYC* on and off states. We observed a variety of high-mannose structures, likely representing immature N-glycans, in both conditions and further identified several low-abundance sialylated bi- and tri-antennary N-glycans that were undetectable in the *MYC* off state (*SI Appendix*, Figs. S6 *A* and *B* and S7 *A* and *B*). Still, the most pronounced effects of *MYC* expression on the glycome were found among the O-glycans.

The elevated levels of disialyl-T in the *MYC* on state suggested this regulation occurred via enzymes or scaffolds underpinning this structure. To identify such elements, we performed RNA-sequencing (RNA-seq) on T-ALL in the *MYC* on and off states (*SI Appendix*, Table S1). We observed significant changes in the expression of over 8,000 genes at a false discovery rate (FDR) of 0.01, and a GO term analysis showed enrichment for genes related to ribosome biogenesis and RNA processing in *MYC* on cells, as has been reported previously (*SI Appendix*, Fig. S8*A* and Table S2) (45–53). To focus our analysis on genes that directly contribute to sialoglycan synthesis, we selected annotated glycogenes exhibiting a log_2_ fold change greater than 1.5 (Fig. 1*F* and *SI Appendix*, Table S3) (54).

Six glycosyltransferases exhibited MYC-dependent increases in expression, including *Fut4*, involved in Lewis^x^ synthesis and metastasis (55, 56), and *Xxylt1*, involved in NOTCH glycosylation (57). However, the most prominent of these MYC-regulated genes was the sialyltransferase *St6galnac4*. The sialyltransferases are a family of twenty enzymes responsible for adding sialic acid through different linkage geometries to underlying glycans (58). We observed that the titrated level of *MYC* expression differentially regulated the expression of various members of this family (Fig. 1*G* and *SI Appendix*, Fig. S8*B*). Importantly, the large increase in MYC-promoted *St6galnac4* expression was accompanied by preferential binding of MYC to the *St6galnac4* promoter (GSE44672; *SI Appendix*, Fig. S8*C*) (35). MYC had no significant effects on the expression of genes involved in sialic acid synthesis, recycling, degradation, or modification (*SI Appendix*, Fig. S8*D*). We performed an additional RNA-seq experiment in which gene expression was measured at various time points after turning *MYC* off to assess whether the emergence of these patterns in sialyltransferase expression correlated temporally with *MYC* (Fig. 1*H* and *SI Appendix*, Table S4). *MYC* transgene expression fell to near baseline levels within 2 h of doxycycline treatment, and within that time frame, *St6galnac4* expression had already decreased by more than 50% (Fig. 1*I*). These data suggest that MYC regulates *St6galnac4*.

Previous work identified ST6GALNAC4 as a disialyl-T synthase that operates by conjugating sialic acid through an α2,6-linkage to sialyl-T (Fig. 1*J*) (59–61). Hence, we next asked whether ST6GALNAC4 is responsible for disialyl-T synthesis in MYC-driven T-ALL. We generated *St6galnac4^−/−^* cells using the CRISPR/Cas9 system. O-glycomics analysis of these knockout cells compared to wild type (WT) revealed a loss of disialyl-T consistent with a role for ST6GALNAC4 as a disialyl-T synthase (Fig. 1*K*). Therefore, MYC-promoted *St6galnac4* expression induces display of the disialyl-T sialoglycan in T-ALL cells.

### ST6GALNAC4 Produces Sialoglycans that Bind to Siglec-E/-7 and Inhibit Macrophage Phagocytosis.

**Fig. 2.**
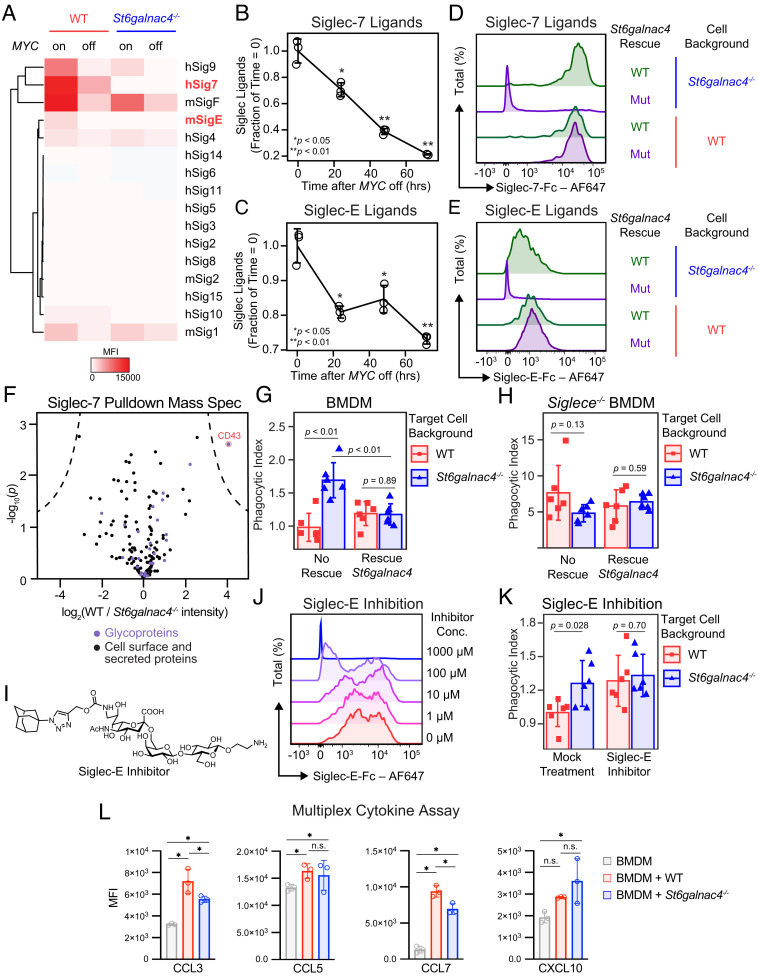
ST6GALNAC4 synthesizes Siglec-E/-7 ligands. (*A*) Heatmap of Siglec binding capacity displayed by control or *St6galnac4^−/−^* T-ALL as measured by flow cytometry following staining with the indicated Siglec-Fc reagent. Cells were treated with either PBS or doxycycline for 48 h to turn off the *MYC* transgene. Siglec-Fc reagents are indicated on the right side of the plot, where “m” indicates murine and “h” indicates human. Bold/red font highlights substantial reductions in the presentation of ligands for human Siglec-7 and murine Siglec-E on *St6galnac4^−/−^* and *MYC* off cells. Plot is representative of two independent experiments. (*B* and *C*) Display of ligands for Siglec-7 (*B*) and Siglec-E (*C*) on murine T-ALL at the indicated time points after turning *MYC* off as quantified by flow cytometry following staining with a Siglec-Fc reagent (n = 3 per time point, two-tailed Student’s *t* test comparing each time point to t = 0 h. *P < 0.05, **P < 0.01, data are representative of two independent experiments). Data are normalized to control, mean ± SD. (*D* and *E*) Representative flow cytometry plots of Siglec-7 (*D*) and Siglec-E (*E*) ligands displayed by WT and *St6galnac4^−/−^* cells reexpressing wild type (WT) or mutant (Mut) *St6galnac4*. Plots are representative of three independent experiments. (*F*) Proteins enriched by immunoprecipitation of T-ALL lysate with Siglec-7-Fc were identified by shotgun proteomics. Annotated cell surface or secreted proteins are displayed on the plot in black, with purple denoting the subset of glycoproteins. The intensity of spectra from WT relative to *St6galnac4^−/−^* T-ALL is displayed [n = 3 per group, significance cutoff by Student’s *t* test with a false discovery rate of 0.0001 and minimum enrichment (S_0_) of 5]. (*G*) Phagocytosis by BMDMs of WT and *St6galnac4^−/−^* T-ALL rescued by transfection with empty vector or *St6galnac4* (n = 6 per group, two-tailed Student’s *t* test). Data are normalized to control (WT, no rescue) and presented as mean ± SD. (*H*) Phagocytosis by *Siglece^−/−^* BMDMs of WT and *St6galnac4^−/−^* T-ALL rescued by transfection with empty vector or *St6galnac4* (n = 6 per group, two-tailed Student’s *t* test). Data are normalized to control (WT target cells, WT BMDMs, no rescue) and presented as mean ± SD. (*I*) Structure of the Siglec-E inhibitor. (*J*) Inhibition of Siglec-E-Fc binding to murine T-ALL by the Siglec-E inhibitor. Plot representative of three independent experiments. (*K*) Phagocytosis by BMDMs of control and *St6galnac4^−/−^* T-ALL in the presence of Siglec-E inhibitor or mock DMSO treatment (n = 6 per group, two-tailed Student’s *t* test). Data are normalized to control (mock-treated WT target cells) and presented as mean ± SD. (*L*) Cytokines released by BMDMs after incubation with anti-Thy1.1 antibody plus WT or *St6galnac4^−/−^* target cells (n = 3 per group, two-way ANOVA using a single family and Tukey’s test for multiple comparisons, * is significant with adjusted *P *< 0.0001). Full multiplex cytokine panel in supplement.

Tumor sialoglycans engage immune cell Siglecs (5–13). We therefore asked whether MYC-promoted disialyl-T engages a Siglec receptor. There are fourteen Siglecs in humans and five homologous proteins in mice. Sialoglycans on T-ALL capable of binding any member of the Siglec family may be detected using Siglec-Fc reagents that comprise the extracellular portion of the Siglec fused to an IgG Fc domain. All commercially available Siglec-Fc reagents were screened against safe targeting control WT and *St6galnac4^−/−^* T-ALL cells in the *MYC* on and off states by flow cytometry. Sialoglycan ligands were detected for human Siglecs-7 and -9, and mouse Siglecs-E and -F, on the surface of *MYC* on WT cells (Fig. 2*A*). In the *MYC* off state, the display of these Siglec ligands decreased, and ligands for Siglec-E and -7 completely disappeared from *St6galnac4^−/−^* cells. Murine Siglec-E is considered the ortholog of human Siglec-7 (62, 63). In *MYC* off cells, ligands for Siglecs-E and -7 steadily decreased over 72 h (Fig. 2 *B* and *C* and *SI Appendix*, Fig. S9).

We examined whether *St6galnac4* is sufficient for the creation of Siglec ligands. We generated WT and *St6galnac4^−/−^* cells reexpressing either a mutant *St6galnac4* predicted to lack catalytic activity or WT *St6galnac4* (*SI Appendix*, Fig. S10*A*). *St6galnac4^−/−^* cells reexpressing WT enzyme recovered the ability to express disialyl-T and bind Siglecs-7 and -E, while *St6galnac4^−/−^* cells expressing the mutant *St6galnac4* did not (Fig. 2 *D* and *E* and *SI Appendix*, Fig. S10 *B*–*D*). Therefore, disialyl-T is produced by *St6galnac4* and is a ligand for mouse Siglec-E and human Siglec-7. Then, we attempted to identify the protein scaffold for disialyl-T by performing immunoprecipitations with Siglec-7 followed by mass spectrometry-based proteomics. By comparing proteins pulled down from WT but not *St6galnac4^−/−^* cells, we found that disialyl-T functions as a ligand for Siglec-7 when linked to the glycoprotein CD43, consistent with a recent report (Fig. 2*F* and *SI Appendix*, Table S5) (64). Notably, *Spn* (the gene encoding CD43) expression was inversely correlated with *MYC* levels in our RNA-seq datasets (*SI Appendix*, Fig. S11 *A* and *B*). Thus, *St6galnac4* synthesizes disialyl-T which functions as a Siglec ligand.

Siglec-E has been reported to modulate macrophage activity (7, 65). Hence, we asked whether the presence of disialyl-T, and thus Siglec-E ligands, on T-ALL decreases phagocytosis by macrophages. T-ALL cells that were labeled with a pH-sensitive red dye to detect internalization and entry into the lysosome were coincubated with bone marrow-derived macrophages (BMDMs). Phagocytosis of *St6galnac4^−/−^* cells in the coculture was higher than that of controls (*SI Appendix*, Fig. S12*A*) and was reversed when the knockout cells were rescued by reexpressing WT *St6galnac4* (Fig. 2*G*). Disialyl-T produced by *St6galnac4* inhibits phagocytosis of T-ALL.

To test if Siglec-E influences macrophage phagocytosis, we performed two studies. First, we generated BMDMs from *Siglece^−/−^* mice. These *Siglece^−/−^* BMDMs phagocytosed tumor cells to a greater extent than their *Siglece*-positive counterparts, but could not discriminate between WT and *St6galnac4^−/−^* target cells (Fig. 2*H* and *SI Appendix*, Fig. S12 *B*–*D*). Second, we synthesized a small molecule selective Siglec-E inhibitor (66) and demonstrated that it blocks the binding of Siglec-E-Fc to target cells (Fig. 2 *I* and *J* and *SI Appendix*, Figs. S13 and S14). The addition of the Siglec-E inhibitor to cocultures both increased macrophage phagocytosis and eliminated the ability of macrophages to discriminate between WT and *St6galnac4^−/−^* target cells (Fig. 2*K*). We conclude that disialyl-T engages Siglec-E and in doing so suppresses macrophage phagocytic activity.

Then, we assessed whether the macrophage secretome is altered by interactions with tumor disialyl-T. Through a Luminex assay, we measured cytokines in BMDM lysate following coculture with T-ALL. BMDMs exposed to *St6galnac4^−/−^* vs. WT T-ALL secreted slightly less CCL3 and CCL7, consistent with a prior report of diminished CCL7 production by Siglec-E knockout BMDMs (67). Production of CXCL10, involved in T and NK cell recruitment, trended upward after exposure to *St6galnac4^-/-^* targets (Fig. 2*L* and *SI Appendix*, Fig. S15). Therefore, tumor disialyl-T alters BMDM phenotype.

### *St6galnac4* Promotes MYC-Driven T-ALL Growth In Vivo.

**Fig. 3.**
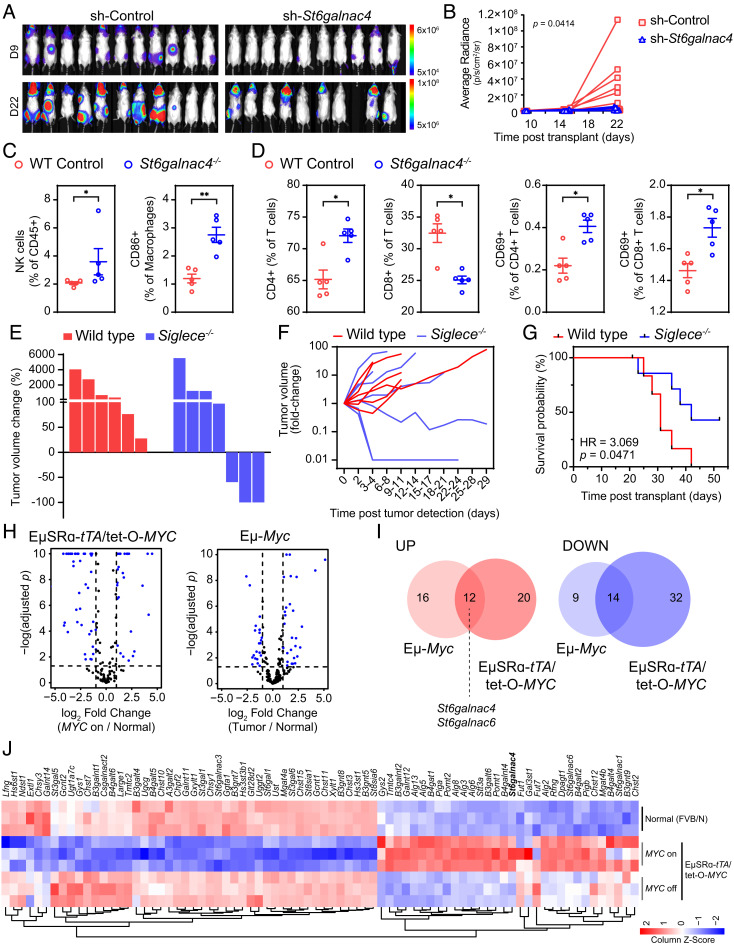
*St6galnac4* promotes tumor growth in vivo*.* (*A*) Bioluminescence imaging of syngeneic WT FVB/N mice transplanted IV with luciferase-labeled MYC-driven T-ALL cells expressing either a *St6galnac4*-specific or control shRNA. Images show tumor burden on day 9 and 22 posttransplantation. (*B*) Tumor growth in mice described in (*A*) was assessed by bioluminescence imaging over time and quantified [n(sh-Control) = 11, n(sh-*St6galnac4^−/−^*) = 9, mixed effects analysis]. (*C* and *D*) Flow cytometric analysis of splenocytes (*C*) and peripheral blood mononuclear cells (*D*) isolated from FVB/N mice 23 d after IV-transplantation of MYC-driven T-ALL expressing either wild type (WT) *St6galnac4* (WT Control, n = 5) or lacking *St6galnac4* expression (*St6galnac4*^−/−^, n = 5). Frequencies of indicated immune cell subsets are shown per mouse (two-tailed Mann–Whitney test, *P < 0.05, **P < 0.01). (*E* and *F*) MYC-driven T-ALL was transplanted subcutaneously into either WT or *Siglece^−/−^* mice. Tumor growth was monitored by caliper measurement. (*G*) Survival of T-ALL bearing WT and *Siglece*^−/−^ mice after undergoing subcutaneous T-ALL transplantation. HR, hazard ratio from the Mantel-Cox test. (*H*) Volcano plots displaying glycogenes in MYC-driven mouse models of Burkitt lymphoma (Eμ-*Myc*) and T-ALL (EμSRα-*tTA*/tet-O-*MYC*). (*I*) Overlap analysis of differentially expressed glycogenes (at least twofold change with FDR < 0.05) from (*H*) (glycogenes up and down: OR = 4.91, *P *= 9.04 × 10^−7^) in mouse models of Burkitt lymphoma (Eμ-*Myc*) and T-ALL (EμSRα-*tTA*/tet-O-*MYC*). (*J*) Heat map representation of glycogenes that are differentially expressed in splenocytes isolated from EμSRα-*tTA*/tet-O-*MYC* (*MYC* on/off) and normal (FVB/N) mice. Expression in normal tissue, in *MYC* on tumor tissue, and upon MYC inactivation-induced tumor regression (*MYC* off) is shown (n = 3 per treatment group).

We asked whether *St6galnac4* and disialyl-T promote tumor growth in vitro and in vivo. To test in vitro growth potential, we observed that *St6galnac4* knockdown T-ALL exhibits a small growth advantage in tissue culture compared to WT T-ALL (*SI Appendix*, Fig. S16 *A* and *B*). To test the effect of *St6galnac4* expression on in vivo tumor growth, T-ALL cells either with or depleted of *St6galnac4* expression were then assessed for their ability to engraft in three different immunological contexts. First, *St6galnac4* knockdown T-ALL exhibited reduced tumor growth when transplanted into immunocompetent, WT FVB/N mice intravenously (Fig. 3 *A* and *B*) and subcutaneously (*SI Appendix*, Fig. S16*C*). Second, intravenous transplantation of T-ALL into immunocompromised *Rag1^−/−^* FVB/N mice showed diminished growth of *St6galnac4*^−/−^ tumors relative to control (*SI Appendix*, Fig. S16 *D* and *E*). Third, intravenous transplantation of T-ALL into immunodeficient NOD/*SCID/IL2Rγ^−/−^* (NSG) mice similarly revealed slowed progression of tumors depleted of *St6galnac4.* This was accompanied by a relative increase in myeloid cells (CD11b+ cells: 14.5% vs. 7.4% in control) (*SI Appendix*, Fig. S16 *F*–*H*). These data indicate that *St6galnac4* promotes the growth and engraftment of T-ALL in vivo.

Next, we examined the effect of tumor-associated *St6galnac4* expression on the host immune environment. We transplanted WT and *St6galnac4^−/−^* T-ALL intravenously in syngeneic hosts and surveyed major immune compartments in the spleen and peripheral blood (Fig. 3 *C* and *D* and *SI Appendix*, Fig. S17 *A* and *B*). NK cell and CD86+ macrophage frequencies were elevated in spleens of mice that received *St6galnac4^−/−^* cells as compared to WT (NK: 2.7% vs. 2.0% of CD45+; CD86+ macrophages: 2.6% vs. 1.2% of CD45+) (Fig. 3*C*). Also, we observed increased frequencies of CD4+ T cells (72.5% vs. 64.7% of T cells) and decreased frequencies of CD8+ T cells (25.1% vs. 32.8% of T cells) in mice that were transplanted with *St6galnac4^−/−^* T-ALL cells compared to WT T-ALL (Fig. 3*D*). This shift toward a higher CD4/CD8 T cell ratio in mice bearing *St6galnac4^−/−^* tumors is similar to the trend observed in the immune system of healthy patients compared to those with cancer (68). Likewise, activated CD4+ and CD8+ T cells as assessed by CD69 positivity were more abundant in mice that received *St6galnac4^−/−^* T-ALL cells as compared to mice that received WT T-ALL (CD69+ CD4+: 0.4% vs. 0.2% of CD4+ T cells; CD69+ CD8+: 1.8% vs. 1.5 % of CD8+ T cells) (Fig. 3*D*). Therefore, T-ALL associated *St6galnac4*, and thus disialyl-T, interferes with myeloid-mediated immunity and T cell activity to promote tumor development in vivo*.* These results are consistent with our observation of reduced phagocytosis of cells expressing *St6galnac4.*

Next, we tested whether the expression of Siglec-E, the receptor for disialyl-T, affects T-ALL progression in vivo by performing T-ALL transplant experiments in Siglec-E knockout mice (69). To enable syngeneic allografts in *Siglece^−/−^* mice, we backcrossed our *MYC*-transgenic T-ALL mouse model to the C57BL/6J background for over 10 generations. We established a new MYC-driven T-ALL tumor-derived cell line from this background. We then transplanted T-ALL cells subcutaneously in WT and *Siglece^−/−^* mice and monitored tumor growth over time. T-ALL cells engrafted in all mice and formed palpable nodules regardless of genetic background, suggesting that Siglec-E expression does not affect tumor engraftment. Interestingly, while all tumors in the WT animals kept growing, tumor regression was seen in three out of seven *Siglece^−/−^* mice (Fig. 3 *E* and *F*). This was accompanied by prolonged survival of *Siglece^−/−^* mice compared to WT mice bearing MYC-driven T-ALL (Fig. 3*G*). Thus, engagement of host Siglec-E promotes MYC-driven T-ALL progression in vivo.

We asked if MYC regulates glycosylation in two autochthonous models that mimic in situ tumorigenesis and engraftment. RNA-seq of the Eμ-*Myc* transgenic mouse model of Burkitt lymphoma (34) and the EμSRα-*tTA*/tet-O-*MYC* model of T-ALL (70) highlighted changes to the expression of several glycogenes upon MYC-driven leukemia and lymphoma development (Fig. 3*H*). Importantly, upregulation of *St6galnac4* is a core component of this glycogene signature and was preserved in both types of MYC-driven leukemia and lymphoma (Fig. 3*I*). The glycogene signature MYC induces upon tumorigenesis is reversible. MYC-directed changes to most glycogenes, including *St6galnac4*, return to normal levels within 4 d of T-ALL regression induced upon MYC inactivation (Fig. 3*J*). We conclude that MYC regulates the expression of glycosylation machinery in general, and *St6galnac4* in particular, during in situ tumorigenesis.

### *MYC* and *ST6GALNAC4* Predict Poor Prognosis in Human Leukemia and Lymphoma.

**Fig. 4.**
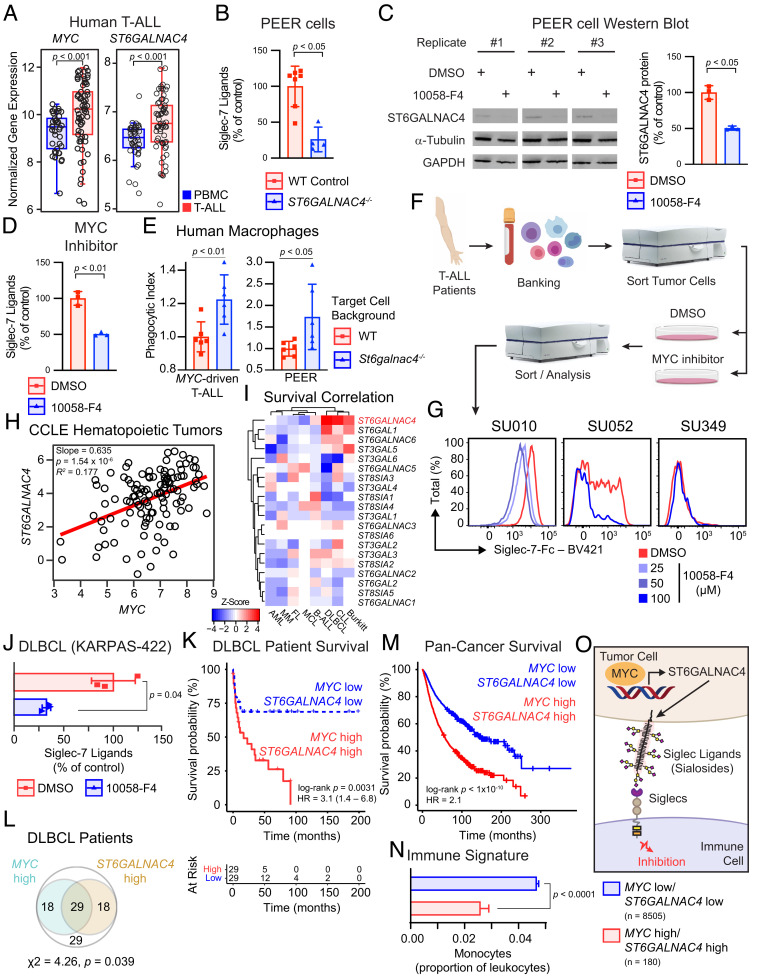
MYC promotes Siglec-7 ligand display on human cancer. (*A*) Summarized gene expression of *MYC* and *ST6GALNAC4* in human T-ALL and peripheral blood mononuclear cells (PBMCs). Data from GSE62156, GSE27562, and GSE49515 were collated, and absolute expression was determined with Gene Expression Commons [n(tumor) = 65, n(control) = 45, two-tailed Mann–Whitney *U* test]. Boxplot shows data quartiles. (*B*) Siglec-7 ligands on PEER cells following knockout of *ST6GALNAC4* [n(control) = 7, n(KO) = 4, two-tailed Mann–Whitney *U* test]. Data presented as mean ± SD. (*C*) Representative Western blot for ST6GALNAC4 in PEER cells following MYC inhibition with 100 μM 10058-F4 for 48 h. Quantification of ST6GALNAC4 band intensity normalized to α-tubulin and WT control (n = 3 per group, two-tailed Student’s *t* test). Data presented as mean ± SD. (*D*) Siglec-7 ligands on PEER cells following pharmacologic MYC inhibition for 48 h by incubation with 100 μM 10058-F4 (n = 3 per group, two-tailed Student’s *t* test). Data presented as mean ± SD. (*E*) Phagocytosis of WT and *St6galnac4^−/−^* murine MYC-driven T-ALL and human PEER cells by human monocyte derived macrophages (n = 6 per group, two-tailed Student’s *t* test). Data are normalized to WT control and presented as mean ± SD. (*F*) Workflow to collect primary T-ALL from patients, treat with a MYC inhibitor (10058-F4), and quantify Siglec-7 ligands. (*G*) Siglec-7 ligands on patient T-ALL liquid biopsies treated with the indicated concentration of 10058-F4 for 48 h. Data represent three donors. (*H*) Correlation of *ST6GALNAC4* and *MYC* mRNA (RNA-seq) gene expression across all hematopoietic tumor samples (n = 106) in the CCLE with *MYC* expression > 3. Correlation determined by least-squares regression. (*I*) Survival Z-score heatmap for each sialyltransferase across each hematopoietic tumor within the PRECOG database. Larger positive Z-Scores (red) indicate that patients with higher expression of the indicated gene exhibit reduced survival. AML, acute myeloid leukemia; MM, multiple myeloma; FL, follicular lymphoma; MCL, mantle cell lymphoma; B-ALL, B cell acute lymphoblastic leukemia; DLBCL, diffuse large B cell lymphoma; CLL, chronic lymphocytic leukemia. (*J*) Siglec-7 ligand display by KARPAS-422 DLBCL cells following treatment with 100 μM 10058-F4 for 48 h (n = 3 per group, two-tailed Student’s *t* test). Data presented as mean ± SD. (*K*) Survival stratified by median *MYC* and *ST6GALNAC4* expression in a cohort of DLBCL patients (GSE4475). HR, hazard ratio from Cox Proportional Hazards model. (*L*) Venn diagram of patients in the same DLBCL cohort, showing individuals that fall into *MYC* high and *ST6GALNAC4* high (greater than median expression) groups (χ^2^-test for independence). (*M*) Pan-cancer overall survival analysis of TCGA data stratifying patients by median *MYC* and *ST6GALNAC4* expression (n = 2,376 per group). HR, hazard ratio from Cox Proportional Hazards model. (*N*) Pan-cancer immune phenotype of TCGA tumors stratified by k-means clustering based on *MYC* and *ST6GALNAC4* expression. Monocyte prevalence was calculated as a fraction of all leukocytes. Data presented as mean ± SEM. (*O*) Model for MYC-driven display of disialyl-T and regulation of the immune response via Siglec engagement.

Direct genetic as well as epistatic activation of MYC through NOTCH1 and other mechanisms drives human T-ALL (22–24, 71). We studied whether MYC also controls glycosylation in human T-ALL. By compiling three existing datasets of over 50 patient samples (72–74), we confirmed elevated *MYC* and accompanying *ST6GALNAC4* expression in human T-ALL (Fig. 4*A* and *SI Appendix*, Table S6). We asked whether ST6GALNAC4 synthesizes sialoglycan ligands for human Siglec-7. Knocking out *ST6GALNAC4* resulted in a sharp reduction in the display of Siglec-7 ligands on the surface of PEER cells, a model human T-ALL cell line (Fig. 4*B* and *SI Appendix*, Fig. S18*A*). We used four different MYC inhibitors, 10058-F4, an inhibitor of MYC/MAX dimerization (75), EN4, a covalent MYC ligand (76), THZ1, a CDK7/9 inhibitor with secondary effects on MYC (77), and lon, a MYC-degrading protease (78) to ask whether MYC regulates the display of Siglec-7 ligands (*SI Appendix*, Fig. S18 *B* and *C*). The pharmacological inactivation of MYC decreased ST6GALNAC4 protein expression (Fig. 4*C*) with an accompanying reduction in Siglec-7-Fc binding (Fig. 4*D*).

We assessed whether tumor *ST6GALNAC4* inhibits phagocytosis by human macrophages similarly to what we observed for murine BMDMs. We differentiated monocyte-derived macrophages from human peripheral blood samples and fed them murine MYC-driven T-ALL and human PEER cells. In both cases, *ST6GALNAC4^−/−^* cells were phagocytosed more readily than WT targets (Fig. 4*E*). We conclude that disialyl-T inhibits phagocytosis by human macrophages.

Next, we asked whether T-ALL liquid biopsies display Siglec-7 ligands susceptible to MYC regulation. We collected samples from patients presenting to the Stanford Hematology Clinic with new onset T-ALL who were naive to therapy. FACS-sorted T-ALL tumor cells were treated with the MYC inhibitor 10058-F4. We detected Siglec-7-Fc binding activity on T-ALL cells from two of three patients. In both cases, MYC inhibition reduced Siglec-7 ligand display (Fig. 4 *F* and *G*). Therefore, MYC promotes display of sialoglycans on primary human T-ALL cells that interact with Siglec-7, consistent with our observations in murine cell lines.

To examine whether MYC regulates *ST6GALNAC4* and Siglec-7 ligand production more generally in human hematopoietic tumors, gene expression data were examined for all hematopoietic tumors contained in the Cancer Cell Line Encyclopedia (CCLE). We found a positive correlation between *MYC* and *ST6GALNAC4* expression (Fig. 4*H*). Through PRECOG (79), we found that high *ST6GALNAC4* expression, among all sialyltransferases, is the strongest predictor of adverse patient outcomes in diffuse large B-cell lymphoma (DLBCL), chronic lymphocytic lymphoma (CLL), and Burkitt lymphoma (BL), which notably all have high MYC activity (Fig. 4*I*) (80). There were no T-ALL data in the PRECOG dataset. Therefore, the survival covariance of all sialyltransferases and *MYC* expression was compared across all tumor types in PRECOG. Indeed, *MYC* and *ST6GALNAC4* had a high covariance. Thus, patients with high *MYC* expression are also likely to have high *ST6GALNAC4* expression (*SI Appendix*, Fig. S19).

The causal relationship of MYC and ST6GALNAC4 activity with Siglec ligand production suggests that their gene expression could serve as a signature of immune inhibitory glycosylation. To test this, we studied DLBCL, an aggressive non-Hodgkin lymphoma characterized by high MYC activity (81). Inhibition of MYC with 10058-F4 caused a 70% reduction in Siglec-7 ligand abundance on KARPAS-422 cells, a model for DLBCL (Fig. 4*J*). In a cohort of DLBCL patients, individuals with combined high *MYC* and high *ST6GALNAC4* expression had strongly decreased overall survival (log-rank *P* = 0.0031) and increased risk of death (HR = 3.1, 95% CI 1.4 to 6.8) (Fig. 4*K*). There was no difference in survival among patients lacking this gene signature (log-rank *P* = 0.58; HR = 1.3, 95% CI 0.50 to 3.6) (*SI Appendix*, Fig. S20). Indeed, patients with either high *MYC* or high *ST6GALNAC4* expression tended to be the same individuals, underscoring the linked effects of these two genes on patient survival (Fig. 4*L*).

Finally, we tested the clinical consequences of combined overexpression of *MYC* and *ST6GALNAC4* in human patients by performing a pan-cancer survival analysis of The Cancer Genome Atlas (TCGA) data. Across all cancers, high expression of *MYC* and *ST6GALNAC4* together is associated with decreased survival (log-rank *P* < 1 × 10^−10^; HR = 2.1) (Fig. 4*M*). Thorsson et al. reported immune profiling data on tumors from patients in the TCGA dataset (82). We analyzed these data to determine whether the *MYC* high*/ST6GALNAC4* high gene signature relates to immune status. Tumors with high *MYC* and high *ST6GALNAC4* had significantly fewer monocytes than tumors lacking this signature (4.7% vs. 2.6%) (Fig. 4*N*). Therefore, the combined overexpression of *MYC* and *ST6GALNAC4* forms a signature of malignant glycosylation associated with a defined immune phenotype. Importantly, pharmacological inhibition of ST6GALNAC4 to block Siglec ligand biosynthesis may be an effective immune therapy strategy for cancers with elevated MYC activity (Fig. 4*O*).

## Discussion

Changes in cell surface glycosylation have been long known as a hallmark of cancer (83), but the functional significance and mechanistic drivers of tumor glycosignatures are poorly understood. We identify a mechanism that drives the production of immune-suppressive tumor sialoglycans. The *MYC* oncogene is one of the most frequently activated cancer-driving genes. Experimentally, suppression of MYC can dramatically reverse tumorigenesis, a phenomenon described as “oncogene addiction.” MYC contributes to tumorigenesis through diverse mechanisms by affecting tumor cell-intrinsic processes and modulating host immune responses. Importantly, the *MYC* oncogene uniquely coordinates key processes by which cancer cells evade the immune system (84). We found that MYC specifically boosts disialyl-T expression and that tumor disialyl-T engages Siglec-E in mice and Siglec-7 in humans, to inhibit macrophages and promote immune evasion in vivo. Thus, our findings highlight MYC-induced immune inhibitory glycosylation as a facet of MYC-mediated immune evasion that contributes to tumorigenesis. These results suggest the potential of the Siglecs and their ligands as targets for immunotherapy of MYC-driven tumors. Further, we reveal disialyl-T as an anti-phagocytic “don’t eat me” signal.

The *MYC* oncogene is activated in a majority of human tumors and is a master regulator of cell-intrinsic and -extrinsic mechanisms of tumorigenesis. MYC activation contributes to cancer by increasing transcriptional activity (32, 33) through interactions with binding partners such as MAX (27–29), MAD (30), MNT (31), and MIZ1 (85), to generally as well as selectively modulate the expression of target genes (34, 35). Via both proximal mechanisms, MYC drives many characteristics of the cancer cell, including elevated biomass accumulation (86), lipogenesis (87, 88), cell proliferation (89), and genomic instability (90), as well as effects on the host microenvironment that can influence metastasis (91), tumor and stromal interactions (85, 92), and immune surveillance and evasion (38). Hence, MYC’s orchestration of the display of immune-modulatory glycans adds another feature to MYC-induced suppression of the anticancer immune response.

Glycan display is a complex process requiring the coordination of metabolic feedstock production with glycosylation machinery. MYC uniquely harmonizes diverse and complex cellular processes, and coordinating immune suppressive glycosylation is likely a key contributor to how MYC drives immune evasion. There are multifactorial potential interactions between T-ALL and stroma that shape the myeloid anti-tumor immune response, including between CD47/SIRPα, PD-L1/PD-1, CD40/CD40L, and CD28/CD80/86. We and others have recently highlighted the role of MYC in modulating the tumor immune microenvironment: MYC regulates the expression of immune checkpoint proteins (38, 40), blocks NK cell-mediated immune surveillance (70, 93), and promotes a chemokine/cytokine profile that favors an immune suppressive tumor microenvironment (94). Our findings reveal glycosylation as a new medium through which MYC manipulates the tumor microenvironment. Further, our results suggest that MYC inactivation may restore the immune response by reversing the contribution of glycosylation to oncogene addiction.

Oncogenes other than MYC have been shown to influence glycosylation, most notably KRAS, EGFR, HER2, BRAF, MEK, and AKT (95). Nevertheless, we note that MYC is frequently activated in cancers driven by other oncogenes (96). For example, RAS cooperates with MYC to promote oncogenesis (97), and RAS activation creates a pool of stabilized, active MYC (98). Our results demonstrate that MYC plays a central role in controlling cellular glycosylation and further outline a specific mechanism by which MYC drives disialyl-T production in hematopoietic tumors such as T-ALL. Differences in glycosylation among various tumors relate to variation in their genetic landscape. In our analysis of patient T-ALL samples, for instance, we identified heterogeneity in Siglec-7 ligand expression potentially related to individual variation in *MYC* and *ST6GALNAC4* expression. Glycogenes are also regulated on several levels in addition to transcription, such as by epigenetic factors including DNA methylation and microRNAs. As a result, cells originating from different tissues express a different suite of glycogenes, and consequently a different set of glycans (*SI Appendix*, Fig. S21*A*). Notably, *ST6GALNAC4* is expressed during lymphocyte development, providing a possible explanation for the strong correlation of *ST6GALNAC4* and *MYC* in T-ALL, DLBCL, and other hematopoietic tumors (*SI Appendix*, Fig. S21 *B* and *C*).

Prior in vitro studies of fibroblasts and colon adenocarcinoma lines found that MYC promotes N-glycan branching (36) and display of the glycan sialyl-Lewis^x^ (37), respectively. The results from these studies were mirrored in the greater diversity, but low abundance, of complex N-glycans in our *MYC* high T-ALL, as well as increased expression of *Fut4*, a fucosyltransferase involved in Lewis^x^ synthesis (55). Although we did not detect many sialyl-Lewis related structures by glycomics, which may be due to differences in cell lineage or differentiation, these data suggest that MYC-directed glycosylation may have important implications for tumor cell adhesion beyond immune evasion. In fact, *N*-acetylgalactosamine-α2,6-sialyltransferases including ST6GALNAC5 (99) and ST6GALNAC4 (100) were previously implicated in metastasis. In the latter case, ST6GALNAC4 was found to “cap” short O-glycans, preventing their elongation by other glycosyltransferases, and in doing so, preserve galectin-3 binding to promote lung cancer metastasis (100). We find that the disialyl-T synthesized by ST6GALNAC4 is itself bioactive and a major contributor to malignancy through its interactions with immune cell Siglecs. We determined that disialyl-T conjugated to the glycoprotein CD43 in particular functions as a ligand for Siglec-7, consistent with recent reports (64, 101, 102). In cancers originating from other tissues, for instance melanoma and neuroblastoma, different glycans, such as those in the glycolipids GD2 (103) and GD3 (104), interact with Siglec-7 (105). Future studies will determine whether MYC might regulate other glycosylation machinery in different tissues of origin to more generally control the display of sialoglycans that engage Siglecs.

Immune cell Siglec receptors and their sialoglycan ligands are emerging as targets for immunotherapy (1, 106, 107). An antibody against Siglec-15 is in clinical trials for the treatment of metastatic solid tumors (NCT03665285) (108), anti-Siglec-9 antibodies are in preclinical development (109), and Siglec-10 has been highlighted as a target for macrophage-directed immunotherapy (5). We previously targeted tumor-associated Siglec ligands by their specific degradation using antibody-sialidase conjugates (10, 11), thereby circumventing the challenge of definitively characterizing Siglec ligand synthesis. This work identifies MYC-driven ST6GALNAC4 as a key node in the synthesis of immune-suppressive glycans. MYC upregulation augments *ST6GALNAC4* expression leading to Siglec-directed immune suppression. Our results suggest that therapeutically targeting MYC could be a general approach to block this immune suppression. Further, inhibition of ST6GALNAC4 activity could function as a potent immune therapy, and its product disialyl-T could be a candidate for checkpoint blockade with an antibody or degradation with an antibody-sialidase conjugate. Finally, MYC-driven cancers may be particularly responsive to such therapies.

## Materials and Methods

Please see *SI Appendix*, *SI Materials and Methods* for a more detailed discussion.

### Cell Lines and Cell Culture.

Murine T cell acute lymphoblastic leukemia (T-ALL) cells were derived from tumors originating in EμSRα-*tTA*/tet-O-*MYC*/FVB/N mice (41). Clonal cell populations were created by single-cell sorting for the subsequent generation of knockout and overexpression cell lines. T-ALL cells for transplant experiments into *Siglece^−/−^* mice were derived from EμSRα-*tTA*/tet-O-*MYC*/C57BL/6J mice (this study). Murine T-ALL was cultured in Roswell Park Memorial Institute (RPMI) 1640 (Gibco) with L-glutamine (Thermo Fisher), 10% tetracycline approved fetal bovine serum (FBS) (Takara), 100 IU/mL penicillin, 100 µg/mL streptomycin, and 50 µM 2-mercaptoethanol. *MYC* expression was reduced to intermediate levels by the addition of 20 pg/mL doxycycline to the media (*MYC* Intermed), turned off by the addition of 500 pg/mL doxycycline (*MYC* off), or maintained by PBS mock treatment (*MYC* on) for 48 h unless stated otherwise. Human cell lines were obtained from the American Type Culture Collection or the DSMZ-German Collection of Microorganisms and Cell Cultures GmbH and cultured according to the Collections’ guidelines. Cells were regularly monitored for *Mycoplasma* infection using PCR-based methods. Cells were cultured in a humidified 5% CO_2_ atmosphere at 37 °C.

### Mouse Models, In Vivo imaging, and Cell Isolation.

MYC-addicted cell lines were derived from EμSRα-*tTA*/tet-O-*MYC/FVB/N* (T-ALL) mice and labeled with firefly luciferase as described in the *SI Appendix*. The minimal number of mice in each group was calculated using the “pwr.t.test” function in the R/pwr package. Then, 2 × 10^6^ luciferase-labeled T-ALL cells were injected intravenously or subcutaneously into 6 to 10-wk-old male FVB/N, *Rag1^−/−^*/FVBN, or NOD/*SCID*/*IL2Rγ^–/–^* (NSG) mice. Engraftment and tumor progression were assessed by caliper measurement or visualized and quantified by bioluminescence imaging (BLI) using an in vivo bioluminescence/optical imaging system (Ami HT, Spectral Instruments Imaging). The luminescence signal was assessed 10 min after intraperitoneal injection of a dose of 3 mg D-Luciferin (Promega) dissolved in PBS per mouse. Imaging was performed under general anesthesia with 2% isoflurane. Image analysis was performed using AmiView software (V1.7.06, Spectral Instruments Imaging), and GraphPad Prism (Version 8.4.1) was used for data visualization and statistical analysis. Spleen tissue was homogenized by passing through a 100-µm mesh and using a 19^1/2^G needle. Erythrocytes were depleted using RBC lysis buffer (BD PharmLyse, BD Biosciences), and washed cells were directly utilized for flow cytometry or cryopreserved (*SI Appendix*, Figs. S22 and S23). No blinding was done while executing the animal experiments. Mouse experiments were approved by the Administrative Panel on Laboratory Animal Care (APLAC) at Stanford University and were carried out in accordance with institutional and national guidelines.

### Flow Cytometry.

Glycans and Siglec ligands were quantified via flow cytometry. Cells were washed with FACS buffer [0.5% bovine serum albumin (BSA) in phosphate-buffered saline (PBS)]. Where indicated, the cells were treated with 100 nM *Vibrio cholerae* sialidase for 30 min at 37 °C. Then, 4 μg/mL Siglec-Fc (R&D), or 10 μg/mL biotinylated *Sambucus nigra* agglutinin (SNA), *Maackia amurensis* agglutinin I (MAA I), or *Maackia amurensis* agglutinin II (MAA II) (Vector Laboratories) was precomplexed with either 8 μg/mL AffiniPure Donkey anti-human IgG Alexa Fluor 647 (Jackson ImmunoResearch), AffiniPure Goat anti-mouse IgG Alexa Fluor 647 (Jackson ImmunoResearch), or 2.5 μg/mL streptavidin Alexa Fluor 647 conjugate (Thermo Fisher Scientific) in FACS buffer for 30 min on ice. Cells were resuspended in the precomplexed lectin-fluorophore conjugate solution at 4 × 10^6^ cells/mL and incubated for 30 min on ice. The cells were then washed twice with FACS buffer. Dead cells were labeled using SytoxBlue (Thermo Fisher Scientific) prior to performing flow cytometry on an LSR II (BD Biosciences). For flow cytometry of murine T-ALL, Purified rat anti-mouse CD16/CD32 (Mouse BD Fc Block, clone 2.4G2) was used for Fc receptor blocking. Cells were then stained with Alexa Fluor 488 rat anti-CD11b (clone M1/70), PE rat anti-mouse CD4 (clone GK1.5), APC rat anti-mouse CD8a (clone 53-6.7) (all from BD Biosciences), and propidium iodide (Invitrogen), and analyzed using BD Accuri C6 Plus (BD Biosciences). For flow cytometric immune phenotyping of splenocytes and peripheral blood mononuclear cells, Fc receptors were blocked using Purified Rat Anti-Mouse CD16/CD32 (Mouse BD Fc Block, clone 2.4G2). Zombie Violet (Biolegend, 1/2,000) was used for live–dead cell discrimination. Cells were stained with the following specific antibodies: PerCP/Cy5.5 Rat anti-mouse CD8 (Tonbo Biosciences, 1/200), PerCP Rat anti-mouse I-A/I-E (Biolegend, 1/100), FITC Rat anti-mouse CD45 (BD, 1/100), BV786 Rat anti-mouse CD11b (BD, 1/300), BV711 Rat anti-mouse F4/80 (Biolegend, 1/100), BV650 Rat anti-mouse CD14 (BD, 1/50), BV605 Hamster anti-mouse CD11c (Biolegend, 1/50), BV510 Rat anti-mouse Ly6C (Biolegend, 1/200), BUV805 Rat anti-mouse CD86 (BD, 1/50), BUV661 Hamster anti-mouse CD3 (BD, 1/100), BUV615 Mouse anti-mouse NK1.1 (BD, 1/100), BUV563 Hamster anti-mouse CD69 (BD, 1/100), BUV496 Rat anti-mouse CD4 (BD, 1/100), APC-Cy7 Rat anti-mouse Ly6G (Tonbo Biosciences, 1/100), Alexa700 Rat anti-mouse CD19 (BD, 1/100), APC Rat anti-mouse CD206 (Biolegend, 1/50), and PE-Cy7 Rat anti-mouse Siglec E (Biolegend, 1/100). Prior to intracellular staining with PE-Daz594 Rat anti-mouse IL-10 (Biolegend, 1/50) cells were fixed and permeabilized using Cyto-Fast™ Fix/Perm Buffer Set. Flow cytometry was performed on a FACSymphony (BD Biosciences).

### Glycomics.

Cells were suspended in a buffer containing 8M urea and 50 mM dithiothreitol. The cell suspension was dialyzed against water using 3 kDa molecular weight cutoff dialysis tubing and stored at −80 °C until further use. For N-glycan release, the cells were treated with PNGase F at 37 °C for 16 h. Released N-glycans were then purified with a C18 column, lyophilized, and stored at −20 °C until further use. For O-glycan release by reductive β elimination, the cell suspension was treated with NaBH_4_ in the presence of NaOH. Released O-glycans were desalted using Dowex 50^+^ resin and lyophilized. Lyophilized glycans were permethylated, extracted with dichloromethane, and analyzed by MALDI-TOF-MS. Additional profiling and fragmentation analysis was conducted using nanospray ionization–mass spectrometry (NSI-FTMS/MS). Permethylated glycans were reconstituted in 50% methanol containing 1 mM NaOH and directly infused into a Fusion Tribrid Orbitrap mass spectrometer (Thermo Fisher). Full mass spectra in addition to an automated “TopN” MS/MS program of the top 300 peaks were collected and fragmented with collision-induced fragmentation (CID). Glycan abundance was calculated using MALDI-TOF-MS spectra as the intensity of m/z peaks corresponding to a particular glycan relative to all other glycans in the spectrum.

### Phagocytosis Assay.

Bone marrow was collected from 6- to 30-wk-old FVB/N mice or from 8- to 10-wk-old C57/BL6 or Siglece*^tm1.2Avrk^*/J^−/−^ mice (Jackson Laboratories, Stock No: 032008). Femurs and tibia were isolated, cleaned of debris, and sterilized by rinsing with ethanol. After removing bone epiphyses, 26-gauge needles were used to flush the bones with PBS and isolate marrow. A uniform cell suspension was then made by passing the marrow through a 19-gauge needle. The cells were then pelleted by centrifugation at 300 g for 5 min, resuspended in media comprising 90% FBS and 10% DMSO, and frozen at −80 °C until use. Bone marrow was differentiated into macrophages by plating in complete media (RPMI with 10% FBS and 1% penicillin/streptomycin) with 25 ng/mL M-CSF (Shenandoah Biotechnology). Complete media and M-CSF were replaced on days 3, 5, and 7. Macrophage differentiation was confirmed by high staining for CD11b and F4/80 and intermediate staining for CD11c. The day prior to performing the phagocytosis assay, macrophages were exposed to trypsin for 5 min and lifted with a cell scraper. Then, 15,000 macrophages were plated per well in a 96-well plate and left to settle overnight. Human macrophages were prepared as described in the supplementary methods. The day of the assay, target cells were labeled by incubation with 0.5 μg/mL pHrodo red (Essen Bioscience) for 1 h. Where indicated, 10 μg/mL of anti-CD90.1 antibody clone 19E12 (BioXCell), mouse IgG2a isotype control (BioXCell), anti-Siglec-E clone 8D2 (Antibodies-Online), rat IgG2b isotype control (Antibodies-Online), or 1 μM Siglec-E ligand was added to the cultures. Then, 60,000 target cells were added to each well of a 96-well plate to give a 4:1 target-to-effector cell ratio. Cells were allowed to settle for 10 min at room temperature prior to live cell imaging on an Incucyte S3 (Essen Bioscience). Phagocytosis was quantified as the area under the curve (AUC) of the integrated red intensity in each well, with a threshold set to exclude extracellular fluorescence. The phagocytosis index was computed as the (AUC of the sample)/(AUC of control group).

### MYC Inhibition in Patient Samples.

Sample collection and study protocols were approved by the Stanford Institutional Review Board (IRB). Samples from treatment naive patients were collected with informed consent and banked by the Stanford Division of Hematology. T-ALL cells were purified from peripheral blood or marrow samples by consulting the patient’s clinical flow phenotype and performing FACS using the following antibodies: anti-CD34:PE clone 582 (BD), anti-CD3:PE-Cy7 clone UCHT1 (BD), anti-CD7:APC clone M-T70 (BD), anti-CD10:APC-Cy7 clone HI10a (Biolegend), anti-CD14:PE-Cy5 clone 61D3 (Invitrogen), anti-CD123:PE-Cy5 clone 6H6 (Biolegend), anti-CD19:PE-Cy5 clone HIB19 (BD), and anti-CD11b:PE-Cy5 clone ICRF44 (BD) (*SI Appendix*, Fig. S24). Purified tumor cells were cultured for 48 h over a monolayer of GFP^+^ hTERT-immortalized mesenchymal stem cells in the presence of the MYC inhibitor 10058-F4 (Sigma). For analysis of Siglec ligands, the cells were resorted and stained with Siglec-Fc probes as described above using a polyclonal goat anti-human IgG:Brilliant Violet 421 (Jackson ImmunoResearch) secondary antibody.

## Supplementary Material

Appendix 01 (PDF)

Dataset S01 (XLSX)

Dataset S02 (XLSX)

Dataset S03 (XLSX)

Dataset S04 (XLSX)

Dataset S05 (XLSX)

Dataset S06 (XLSX)

## Data Availability

Raw sequencing datasets are available in the Gene Expression Omnibus at GSE222939 (110). Processed RNA-seq data are available (*SI Appendix*, Tables S1 and S4). Links to download other gene expression datasets analyzed in this study are included in the relevant section of the supplemental methods. All study data are available in the article or *SI Appendix*.
