# Supplementary material for: MYC-driven synthesis of Siglec ligands is a glycoimmune checkpoint
Source: Proc Natl Acad Sci U S A. 2023 Mar 10;120(11):e2215376120. doi: 10.1073/pnas.2215376120 (PMC10089186; doi:10.1073/pnas.2215376120)
Supplement: Supplementary file 1 — Appendix 01 (PDF) [file pnas.2215376120.sapp.pdf]

PNAS
www.pnas.org

Supplementary Information for:
MYC-driven synthesis of Siglec ligands is a glycoimmune checkpoint

Benjamin A. H. Smith†, Anja Deutzmann†, Kristina M. Correa, Corleone S. Delaveris, Renumathy Dhanasekaran, Christopher G. Dove, Delaney K. Sullivan, Simon Wisnovsky, Jessica C. Stark, John V. Pluvinage, Srividya Swaminathan, Nicholas M. Riley, Anand Rajan, Ravindra Majeti, Dean W. Felsher* and Carolyn R. Bertozzi*

† These authors contributed equally to this work.

* Carolyn R. Bertozzi and Dean W. Felsher are co-corresponding authors.

Email: bertozzi@stanford.edu and dfelsher@stanford.edu.

This PDF file includes:

Supplemental Materials and Methods
Figs. S1 to S24
Supplementary Table Legends
SI References

Other supplementary materials for this manuscript include the following:

Tables S1 to S6

Supplemental Materials and Methods

Statistics. Measurements were made on independent samples unless otherwise noted. Nonparametric tests were used when data could not be assumed to follow a normal distribution or verified to do so by plotting. Test statistics and effect sizes were calculated in R(1) and Prism (GraphPad). General data wrangling was performed with the tidyverse(2) package in R.

Reagents and general synthetic methods. Unless stated otherwise, reactions were conducted in oven-dried glassware under an atmosphere of nitrogen using anhydrous solvents. Tetrahydrofuran (THF) and dichloromethane (DCM) were purified by first purging with dry nitrogen, followed by passage through columns of activated alumina. Deionized water was purified to 18 MΩ-cm using a Millipore Milli-Q Biocel A10 purification unit. All commercially obtained reagents were used as received without further purification unless otherwise stated. Molecular sieves (Sigma-Aldrich, 688363) were flame-dried under vacuum and used immediately after cooling. Thin layer chromatography was conducted on SiliCycle silica plates (TLG R10011B-624), detected using multiband UV-absorption (254 – 365 nm), and visualized using a combination of UV, anisaldehyde, $KMnO_4$, H_2SO_4, ninhydrin, and phosphomolybdic acid staining. Column chromatography was done with Biotage SNAP KP-Sil (FSK0-1107) and an Isolera Prime ACI automated fraction collector from Biotage. NMR spectra were obtained on Varian spectrometers at room temperature at the Stanford Department of Chemistry NMR Facility.

Retro- and lentiviral transduction. Phoenix cells containing the plasmids pHIT60 (gag-pol) and pHIT123 (ecotropic envelope) (provided by G.P. Nolan) were cultured in regular growth medium consisting of high-glucose DMEM (Invitrogen) with GlutaMAX containing 10% FBS, 0.1 mM nonessential amino acids, 100 IU/ml penicillin, 100 µg/ml streptomycin, 25 mM HEPES (pH 7.2), and 1 mM sodium pyruvate. Retroviral particles to infect murine cells were generated by transfecting Phoenix cells with the retroviral luciferase expression plasmid pMSCV-neo-Luc2(3) using Lipofectamine 2000 in serum-free Opti-MEM media (both Invitrogen). An equal volume of regular growth media supplemented with additional 10% FBS was added 2-3 hours after transfection. After 16 h, media was replaced with regular growth medium containing 10 mM sodium butyrate. After 8 h of incubation, the medium was again changed to regular growth medium. Viral supernatants were collected 24 h later, filtered (0.45- µm), and then centrifuged at 2,000*g* for 90 min on 50 µg/ml RetroNectin-coated non–tissue-culture-treated six-well plates. Supernatant was removed and target cells plated into virus-coated plates. Luciferase-expressing cells were selected for with Neomycin (400 µg/ml) starting two days after transduction.

Lentiviral particles to infect murine cells were produced by co-transfecting HEK 293FT cells with the plasmids psPAX2 (gag-pol; Didier Trono, Addgene 12260), pMD2.G (VSV-G envelope; Didier Trono, Addgene 12259), and a pLKO.1 plasmid containing *St6galnac4*-specific or scrambled control shRNA. Four different shRNAs (all from Sigma MISSION) were tested for

knockdown efficiency (TRCN0000110470: CCGGCCCTGGAACTTGGGATTTGATCTCGAGATC AAATCCCAAGTTCCAGGGTTTTTG, TRCN0000110471: CCGGGCGCAACTACTCACACTATTT CTCGAGAAATAGTGTGAGTAGTTGCGCTTTTTG, TRCN0000110472: CCGGGCTAGATGAGT GTCAGATGTACTCGAGTACATCTGACACTCATCTAGCTTTTTG, TRCN0000110473: CCGGCC GGAGACAATCAGGCTCCTTCTCGAGAAGGAGCCTGATTGTCTCCGGTTTTTG, TRCN000011 0474: CCGGGTACACCTTCACTGAACGCATCTCGAGATGCGTTCAGTGAAGGTGTACTTTTTG). Transfections were performed using Lipofectamine 2000 (Invitrogen) with Opti-MEM media (Invitrogen). Transfected 293FT cells were cultivated in high-glucose DMEM (Invitrogen) with GlutaMAX as described above. Culture supernatants were concentrated with Lenti-X™ Concentrator (Clontech) according to the manufacturer's recommendation. Target cells were transduced with concentrated virus particles by spin infection in target cell culture media supplemented with polybrene (8 µg/ml).

CRISPR/Cas9 cell line engineering. Knockout cell lines were generated via electroporation of sgRNA/Cas9 nucleoprotein complexes or lentiviral transfection of the lentiCRISPR v2 plasmid, provided as a gift from Feng Zhang (Addgene plasmid # 52961 ; http://n2t.net/addgene:52961 ; RRID:Addgene_52961)(4). To knock out *St6galnac4* in murine T-ALL, 6 µL of a synthetic guide RNA (Synthego) at a concentration of 30 pM with the sequence UGCACAGUGUGAGGAGCAGA was complexed with 1 µL of Cas9-2NLS (Synthego) at 20 pM in 18 uL nucleofection buffer [120 mg/mL KH_2PO_4, 1.2 mg/mL $NaHCO_3$, 0.4 mg/mL glucose, 4 mg/mL ATP disodium salt, 2.4 g/mL $MgCl_2 \cdot 6H_2O$, pH 7.4] for 10 minutes at room temperature. Then, 100,000 cells were added to the complexes, the volume was brought up to 100 µL using nucleofection buffer, and the mixture was transferred to a nucleocuvette. Nucleofection was performed using a Nucleofector II (Amaxa Biosystems) and the program X-001. Cells were then transferred to 1 mL of complete media in a 24-well plate for recovery.

Quantitative RT-PCR. *St6galnac4* mRNA expression was assessed after puromycin (2 µg/ml) selection by quantitative real-time PCR. RNA was extracted using RNeasy Plus Mini Kit (Qiagen), and reverse transcription PCR was performed using SuperScript III First-Strand Synthesis System for RT-PCR (Invitrogen) following manufacturer's instructions. Quantitative real-time PCR was carried out using specific primers (*St6galnac4*: F: AGCCTCTTATCCGAGAACTGT, R: GGCCTGAACCCAGCATCTG; *Ubc*: F: AGCCCAGTGTTACCACCAAG, R: CCTTGTGCTTGTTCTTGGGT), SYBR GreenER mix (Invitrogen) according to standard PCR conditions and an ABI 7900HT real-time PCR system (Applied Biosystems).

Sialyltransferase rescue expression. *St6galnac4* expression rescue plasmids were based on the pSBbi-GB backbone, provided as a gift from Eric Kowarz (Addgene plasmid # 60520;

http://n2t.net/addgene:60520; RRID:Addgene_60520)(5). Sequences corresponding to WT *St6galnac4* or mutant *St6galnac4* (C76A, R102A, C225A, H274A, and E279A), predicted to be catalytically inactive, were inserted into pSBbi-GB via In-Fusion cloning (Takara). Cells were then transfected with plasmids packaged with Lipofectamine 3000 (Thermo Fisher). Successfully transfected cells were selected as blasticidin-resistant and GFP-positive.

Western blotting. Cell lysates were prepared by lysis in RIPA buffer with the cOmplete protease inhibitor cocktail (Thermo Fisher Scientific). Samples were then centrifuged at 13,000*g* for 10 min. Protein concentration in the supernatant was determined with a Bradford assay (Bio-Rad). Between 5 and 20 µg of protein from each sample was mixed with SDS loading buffer, boiled for 5 min at 95 °C, and then run on an SDS–PAGE gel. Samples were then transferred to a nitrocellulose membrane (Bio-Rad), which was blocked with Odyssey Blocking Buffer (LI-COR) for 1 hour. The membrane was then stained at room temperature for 1 hour with primary antibodies at the indicated concentrations: anti-MYC (1:10,000, clone Y69, Abcam #ab32072), anti-α-Tubulin (1:10,000; clone DM1A, Sigma #T9026), anti-ST6GALNAC4 (1:500, clone PA5-43295, Invitrogen). The membrane was washed with PBS containing 0.1% Tween-20, and stained with an IRDye conjugated secondary antibody (1:15,000, LI-COR) before imaging on the Odyssey CLx (LI-COR).

Periodate-aminooxy ligation. Sialic acids were labeled via mild periodate oxidation and detected as previously described(6). In brief, $4x10^5$ cells were plated in each well of a 96 well plate. Cells were washed three times with PBS, and then resuspended in 1 mM $NaIO_4$ in PBS at pH 7.4 for 30 min on ice. Cells were washed once and each well was resuspended in 100 µL of FACS buffer (0.5% bovine serum albumin (BSA) in phosphate buffered saline (PBS)). 50 µL of 5 mM glycerol was added to each well, mixed by pipetting, and incubated for 10 minutes on ice. Cells were then pelleted and washed twice with 5% FBS in PBS at pH 6.7. Aldehydes were then labeled by resuspending each well of cells in 200 µL of a solution comprising 0.1 mM aminooxy biotin (Thermo Fisher), 10 mM aniline, and 5% FBS in PBS at pH 6.7, and incubating for 90 min on ice. Cells were washed twice with 5% FBS in PBS at pH 6.7, and resuspended in FACS buffer containing 3.2 µg/mL AlexaFluor 647-streptavidin (Thermo Fisher), for 30 min. Finally, cells were washed twice with FACS buffer, dead cells were labeled with SytoxBlue, and flow cytometry was performed on an LSR II.

DMB derivatization of sialic acids. Sialic acids were released and labeled with 1,2-diamino-4,5-methylenedioxybenzene (DMB) as previously described(7). In brief, cells were washed in Dulbecco's PBS (DPBS), resuspended in hypotonic lysis buffer [10 mM Tris HCl at pH 7.3, 10 mM $MgCl_2$, 1 mM EDTA, and 1 mM EGTA] and then lysed by extrusion through a 25-gauge

needle thirty times. After lyophilization overnight, the lysate was resuspended in 100 µL of 2M acetic acid and incubated at 80 ºC for two hours. Then 40 µL of DMB buffer [7 mM DMB, 750 mM β-mercaptoethanol, 18 mM Na_2SO_4, and 1.4 M acetic acid] was added to the samples and incubated at 50 ºC for two hours. Samples were neutralized by the addition of 5 µL of 0.2 M NaOH. Samples were passed through a 10 kDa molecular weight cutoff filter by centrifugation. 10 µL of filtrate was diluted into 90 uL of water, and 20 uL of this solution was analyzed by reverse phase HPLC using a Poroshell 120, EC-C18 column (Agilent). The following gradient of acetonitrile in water at a flow rate of 1 mL per minute was used: T(0 min) 2%; T(2 min) 2%; T(5 min) 5%; T(25 min) 10%; T(30 min) 50%; T(31 min) 100%; T(40 min) 100%; T(41 min) 2%; T(45 min) 2%. DMB-conjugated sialic acids were detected by fluorescence excitation at 373 nm and emission at 448 nm. Sample peak areas and elution times were compared to Neu5Ac (Jülich Fine Chemicals) and the Glyko Sialic Acid Reference Panel (Prozyme).

Genome-wide gene expression analysis. RNA was isolated using an RNeasy kit (Qiagen) and assessed for degradation using a 2100 Bioanalyzer (Agilent). cDNA was generated using the SMART-seq v4 kit (Takara) with oligo(dT) priming, and libraries were prepared with the Nextera XT Library Prep Kit (Illumina). Libraries were pooled and sequenced on a HiSeq-4000 (Illumina). For the MYC inactivation time course experiments, polyA-selected libraries were prepared by the BGI Group using the BGISeq-500 library construction protocol and were sequenced as paired-end reads of 100 base pairs on a BGISeq-500 platform. Reads were demultiplexed with bcl2fastq, mapped to the mm10 reference genome with STAR(8) using the GENCODE vM16 comprehensive annotations, and then counted using featureCounts from Rsubread(9). Differential gene expression analysis was performed using DESeq2(10) with the FDR set to 0.01. *P* values were adjusted for multiple hypothesis testing. Gene annotations were accessed with biomaRt(11), and GO term analysis was conducted with topGO(12) after setting a $\log_2$ fold-change expression threshold of 1. Mouse glycogene annotations were pulled by referencing the GlycoGene Database(13) through GlyCosmos (https://glycosmos.org/). Glycogene expression was also examined in murine Burkitt's lymphoma (GSE51008)(14) and T-ALL (GSE106078)(15).

Siglec immunoprecipitation and proteomics sample preparation. Murine MYC-driven T-ALL control or *St6galnac4*$^{-/-}$ cell pellets were resuspended in lysis buffer [1x PBS + 0.1% NP-40 (Abcam) + 1x Halt protease inhibitor cocktail (Thermo Fisher Scientific)] and lysed via sonication. Lysates were clarified via centrifugation at 20,000*g* for 10 min at 4 ºC and lysate concentration was normalized to 1 mg/mL total protein in lysis buffer. Immunoprecipitation samples were prepared in parallel with untreated lysate and lysate pre-treated with 100 nM *V. cholerae* sialidase for 3 h at 37 ºC. During sialidase treatment, Siglec-Fc (R&D Biosystems) was precomplexed on Protein G beads (Invitrogen). For each sample, 50 µL Protein G beads were aliquoted into a 1.5

mL microcentrifuge tube and washed once with 250 µL PBS. Siglec-Fc was added to beads (250 µL of 20 µg/mL Siglec-Fc stock in 1x PBS) and precomplexed via incubation for 1 h at room temperature with rotation. After 1 h, beads were washed once with 250 µL PBS to remove unbound Siglec-Fc. Following sialidase treatment, 500 µL sialidase treated or untreated lysate was added to the precomplexed Siglec-Fc magnetic beads and incubated overnight at 4 ºC with rotation. Immunoprecipitations were performed in biological triplicate.

After overnight incubation, beads were washed twice with 200 µL lysis buffer and three times with 200 µL mass spectrometry grade 50 mM triethylammonium bicarbonate (TEAB; Thermo Fisher Scientific). Immunoprecipitated ligands were eluted from beads by boiling in 50 µL 50 mM TEAB + 0.05% Rapigest (Waters) for 10 min at 100 ºC and eluate was collected. Beads were washed with an additional 50 µL of 50 mM TEAB + 0.05% Rapigest, which was combined with the first eluate fraction. Eluates were reduced via addition of 5 mM 1,4-dithiothreitol (DTT; Sigma) and incubation at 60 ºC for 30 min with shaking. Samples were then alkylated via addition of 10 mM iodoacetamide (Sigma) and incubated at room temperature for 30 min with shaking. Samples were further digested with 1 µg sequencing grade trypsin (Promega) via overnight incubation at 37 ºC with shaking. Samples were acidified via addition of mass spectrometry grade 2% formic acid (FA; Sigma) and incubated at 37 ºC for 30 min with shaking. Samples were then dried in a vacuum concentrator and cleaned up using Strata-X columns (Phenomenex). Strata-X columns were activated with 1 mL mass spectrometry grade acetonitrile (ACN; Sigma). Dried samples were then resuspended in 1 mL of 0.1% FA in mass spectrometry grade water (Sigma) and loaded onto activated Strata-X columns. Columns were washed with 1 mL of 0.1% FA and samples were eluted with 400 µL of 0.1% FA in 80% ACN in water. Eluates were dried in a vacuum centrifuge and resuspended in 10 µL of 0.1% FA. Peptide concentrations were measured via A205 on a Nanodrop (Thermo Fisher Scientific) and normalized to 1 mg/mL in 0.1% FA. Immunoprecipitated ligands were identified via LC-MS/MS analysis.

Mass spectrometry identification of Siglec ligands. For each immunoprecipitation sample, 1 ug was injected for LC-MS/MS analysis. Peptides were separated over a 25 cm EasySpray reverse-phase LC column (75 µm inner diameter packed with 2 µm, 100 Å, PepMap C18 particles, Thermo Fisher Scientific). The mobile phases (A: water with 0.2% FA and B: acetonitrile with 0.2% FA) were driven and controlled by a Dionex Ultimate 3000 RPLC nano system (Thermo Fisher Scientific). An integrated loading pump was used to load peptides onto a trap column (Acclaim PepMap 100 C18, 5 µm particles, 20 mm length, Thermo Fisher Scientific) at 5 µL/minute, which was put in line with the analytical column 5 minutes into the gradient. The gradient was held at 0% B for the first 6 minutes of the analysis, followed by an increase from 0% to 5% B from 6 to 7 minutes, an increase from 5 to 25% B from 7 to 66 minutes, an increase from 25% to 90% from 66 to 70 minutes, isocratic flow at 90% B from 70 to 75 minutes, and re-

equilibration at 0% B for 15 minutes for a total analysis time of 90 minutes. Eluted peptides were analyzed on an Orbitrap Fusion Tribrid MS system (Thermo Fisher Scientific). Precursors were ionized using an EASY-Spray ionization source (Thermo Fisher Scientific) held at +2.2 kV relative to ground, the column was held at 40 °C, and the inlet capillary temperature was held at 275 °C. Survey scans of peptide precursors were collected in the Orbitrap from 350-1350 Th with an AGC target of 1,000,000, a maximum injection time of 50 ms, RF lens at 60%, and a resolution of 60,000 at 200 m/z. Monoisotopic precursor selection was enabled for peptide isotopic distributions, and precursors of z = 2-5 were selected for data-dependent MS/MS scans for 2 seconds of cycle time. Dynamic exclusion was set to exclude precursors after being selected once for an exclusion time of 30 seconds with a ±10 ppm window set around the precursor monoisotope. An isolation window of 1 Th was used to select precursor ions with the quadrupole, and precursors were fragmented using a normalized HCD collision energy of 30. MS/MS scans were collected with an AGC target of 100,000 ions, with a maximum accumulation time of 54 ms and an Orbitrap resolution of 30,000 at 200 m/z. The same method was used for both untreated and sialidase treated samples. Raw data were processed with MaxQuant(16) version 1.6.2.10 and tandem mass spectra were searched with the Andromeda(17) search algorithm. 20 ppm, 4.5 ppm, and 20 ppm were used for first search MS1 tolerance, main search MS1 tolerance, and MS2 product ion tolerance, respectively. Oxidized methionine and deamidated asparagine were set as variable modifications, and carbamidomethylation of cysteine was set as a fixed modification. Cleavage specificity was set to Trypsin/P with 2 missed cleavages allowed. Peptide spectral matches (PSMs) were made against a mouse protein database downloaded from Uniprot. Peptides were filtered to a 1% false discovery rate (FDR) using a target-decoy approach(18), and a 1% protein FDR was applied. Proteins were quantified and normalized using MaxLFQ, and the match between runs feature was enabled. Label free intensity values were log2 transformed and plotted using Perseus version 1.6.2.2(19). Identified proteins were filtered to plot those that are annotated as localized on the cell surface or secreted, but not cytoplasmic (via UniProt annotations). Significance cutoffs for volcano plots were determined using student’s *t* test with a false discovery rate of 0.0001 and minimum enrichment (S_0) of 5.

Human macrophage preparation. Peripheral blood mononuclear cells (PBMCs) were isolated from Leukoreduction System (LRS) chambers from the Stanford Blood Bank using SepMate tubes (Stemcell) and the manufacturer’s instructions. In brief, 15 mL of density gradient medium was added to a 50 mL SepMate tube. The sample was diluted 1:1 in 2% FBS in PBS, added to the SepMate tube and centrifuged at 1200 *g* for 10 minutes. The sample was eluted into a new 50 mL tube and washed twice with 2% FBS in PBS. A quantity of 4 x 10^8 PBMCs was plated per T75 tissue culture flask in 20 mL of macrophage medium comprising IMDM with 10% Human AB Serum (Gemini). On day 3, 10 mL of macrophage medium was added to each plate. On day 5,

the macrophage medium was removed and replaced with 20 mL fresh medium. Cells were used in phagocytosis experiments on days 8-10. On the day prior to the phagocytosis assay, macrophages were collected with trypsinization and gentle scraping with a cell lifter. The macrophages were rinsed with complete media and then plated in a 96-well plate at a concentration of 20,000 cells per well in a volume of 100 uL of complete media. Phagocytosis assays were completed as described above.

Multiplex cytokine assay. BMDMs were cultured as described in the main manuscript methods in RPMI with 10% FBS and 25 ng/mL M-CSF. On day 7 of culture, 1 x 10^5 macrophages were plated per well in a 12-well plate in 500 uL medium. 2 x 10^5 tumor cells were added to each well, and where indicated, anti-Thy1.1 (clone 19E12, BioXCell) was added to a final concentration of 10 ug/mL. Following 24 hours of coculture, macrophages were isolated by vigorous rinsing with PBS. The macrophages were then lysed directly by treating with 200 uL RIPA (140 mM NaCl, 1% Triton X-100, 0.5% sodium deoxycholate, 0.1% sodium dodecyl sulfate, 50 mM Tris, pH 8.0) with a cOmplete protease inhibitor tablet (Roche) and incubating on ice for 30 minutes. The lysate was transferred to a low protein-binding polypropylene microcentrifuge tube (Eppendorf) and clarified by centrifuging at 10,000*g* for 10 minutes. The supernatant was stored at -80 ºC prior to analysis with the 48-Plex Mouse ProcartaPlex Panel (Invitrogen) per the manufacturer’s instructions.

Patient survival analyses and bioinformatics. T-ALL patient (GSE62516)(20) and PBMC (GSE27562 and GSE49515)(21, 22) gene expression datasets were normalized and compared using the Gene Expression Commons portal(23). The *MYC* probe used in the analysis was 202431_s_at, while the *ST6GALNAC4* probe was 220937_s_at. Cross cancer survival meta-analyses were performed using data from PRECOG(24). Survival analyses and Cox Proportional Hazards modeling of DLBCL (GSE4475)(25) were conducted using the survival, survminer and survivalAnalysis packages in R. Analyses focused on overall survival and the indicated genetic signatures.

The Cancer Genome Atlas (TCGA) pan-cancer analysis. We examined the impact of high *MYC* and *ST6GALNAC4* expression in human cancers using the pan-cancer TCGA data from 9502 human patients with 33 different cancers using the interactive server GEPIA(26). Kaplan-Meier analysis was performed for survival analysis. A log-rank test was used to compare survival between patients with the highest quartile of combined *MYC* and *ST6GALNAC4* overexpression (n=2376) with the lowest quartile of *MYC* and *ST6GALNAC4* expression (n=2376). Pan-cancer RNA-seq data was downloaded from the GDC data portal (https://portal.gdc.cancer.gov/) on May 24, 2020. The cellular fraction estimates of the deconvoluted immune subset data for the pan cancer TCGA cohort was downloaded from the GDC portal (https://gdc.cancer.gov/about-

data/publications/panimmune) on May 21, 2020. We evaluated the effect of MYC and ST6GALNAC4 on the immune microenvironment of human tumors via CIBERSORT analysis of the human pan-cancer TCGA study(27). K-means clustering was used to stratify patients into two groups based on *MYC* and *ST6GALNAC4* expression. Welch's *t* test was used to compare the proportion of different immune subsets in *MYC* high / *ST6GALNAC4* high tumors (n=1780) versus *MYC* low / *ST6GALNAC4* low tumors (n=9323). Lastly, the correlation between *MYC* and *ST6GALNAC4* expression was evaluated using a Spearman correlation test.

Pan-cancer *MYC* and sialyltransferase correlation. Human RNA-seq data from TCGA was downloaded from the UCSC Xena Toil project(28), which provides the log2-transformed RSEM expected counts(29) normalized using DESeq2(10). The R library, ComBat(30), was used for batch effect correction in TCGA data using biospecimen batch identifier data obtained from the National Cancer Institute's Genomic Data Commons (GDC) data portal. The final processed TCGA gene expression data is available at https://github.com/Yenaled/felsher/releases/download/felsher/TCGA.processed.tumors_corrected.tsv.gz and Pearson's correlations (computed in R) are available at https://github.com/Yenaled/felsher/blob/master/output/tcga_correlation/myc_correlations.csv.gz.

Protein expression and purification for chemoenzymatic syntheses. Expression plasmids for Pd26ST and NmCSS were a kind gift from the lab of Xi Chen. Pd26ST and NmCSS were expressed in BL21(DE3) E. coli and isolated as previously described(31). In brief, starter cultures were grown in 5 mL LB with antibiotic selection and then 1 L cultures were inoculated from the starter culture. Cultures were grown at 37 °C to an OD600 of 0.8-1.0, at which point expression was induced with IPTG (0.1 mM) and cultures were grown overnight at 20 °C with shaking at 220 rpm. After 24 hours, cells were pelleted by centrifugation and lysed in buffer (250 mM Tris HCl, 0.5 M NaCl, 20 mM imidazole, 0.1% Triton X-100) supplemented with protease inhibitor cocktail (Sigma Aldrich) (one tablet per 40 mL) and DNAse I (Thermo Fisher) (10 µL per 40 mL). Cells were lysed using a dounce homogenizer followed by French press. Lysates were clarified by centrifugation and purified on HisTRAP columns (GE Life Sciences) using a gradient of 20 mM to 200 mM imidazole on an AKTA FPLC. Fraction purity was determined by SDS-PAGE and pure fractions were combined, purified by dialysis against storage buffer (50 mM Tris HCl, 250 mM NaCl, 10% glycerol), aliquoted, and flash-frozen in liquid nitrogen for storage in a -80 °C freezer.

Organic synthesis. The Siglec-E inhibitor was synthesized using previously described methods **(Fig. S13 and S14)**(32). In brief, steps for synthesis of each intermediate are as follows:

9-N'-propargylcarbamyl-9-deoxy-5-N-acetyl neuraminic acid. The known 9-amino-9-deoxy neuraminic acid (380 mg, 1.2 mmol) was dissolved in methanol (22 mL, 0.05 M) and *N*,*N*-

diisopropylethylamine (~0.5 mL) was added until a basic pH ~9 was reached. Propargyl chloroformate (0.15 mL, 1.5 mmol, 1.2 eq.) was added to this solution over a period of 5 minutes. The reaction was stirred for 6 hours at room temperature. The solvent was then evaporated in vacuum and the residue was purified on a Biogel P2 column (Biorad, 1504118) to afford product as a white foam. Spectral data were in accordance with the literature(32). For use in chemoenzymatic reactions, products were dissolved in water to 180 mM, aliquoted, and frozen.

(9-N-propargylcarbamyl)-sialyl-α-2,6'-lactose β-ethylamine. A solution of (2-amino)ethyl lactose (150 mg, 0.39 mmol), 9-N'-propargylcarbamyl-9-deoxy-5-N-acetyl neuraminic acid (2.6 mL of 180 mM, 0.47 mmol, 1.2 equiv.), and cytidine triphosphate (270 mg, 0.51 mmol, 1.2 equiv.) in a 100 mM Tris buffer containing 20 mM $MgCl_2$ (18 mL, 20 mM lactose) was adjusted to pH 8.5 with 2 N NaOH. Freshly thawed stocks of Pd26ST (to 0.3 mg/mL) and NmCSS (to 0.2 mg/mL) were added to the reaction mixture. Conversion was monitored by LCMS. When the reaction mixture reached peak conversion, it was pelleted by centrifugation (3700*g*, 5 min) and the supernatant was decanted and frozen in liquid nitrogen. The solid was lyophilized. The reaction mixture was resuspended in ddH_2O with 0.1% TFA. The insoluble material was removed by centrifugation (3000*g*, 30 min) and the supernatant was purified via reverse phase flash chromatography (C18, 0 to 15% MeCN in ddH_2O with 0.1% TFA). Product-containing fractions were identified by UV-Vis and lyophilized to afford product as a fluffy white solid (13 mg, 4%). Spectral data were consistent with the literature.(32)

Siglec-E inhibitor. To a solution of intermediate sialoside (13 mg, 17 µmol) and sodium ascorbate (4.0 mg, 20 µmol, 1.2 equiv.) in water (114 µL, 180 mM) was added a solution of 200 mM adamantyl azide in *tert*-butanol (171 µL, 34 µmol, 2.0 equiv.). To this suspension was added a 50 mM solution of BTTAA (137 µL, 6.8 µmol, 0.4 equiv.) followed by a solution of 50 mM $CuSO_4$ (68 µL, 3.4 µmol, 0.2 equiv.). The reaction was stirred overnight. The reaction mixture was lyophilized, redissolved in 1 mL of 0.1% TFA in water, pelleted by centrifugation (16,000 rcf, 15 min), and purified by reverse phase chromatography (C18, 10 to 40% MeCN in ddH2O with 0.1% TFA). Product-containing fractions were determined by LCMS, combined, and lyophilized to afford the Siglec-E inhibitor (8.5 mg, 53%). Spectral data were consistent with the literature.(32)

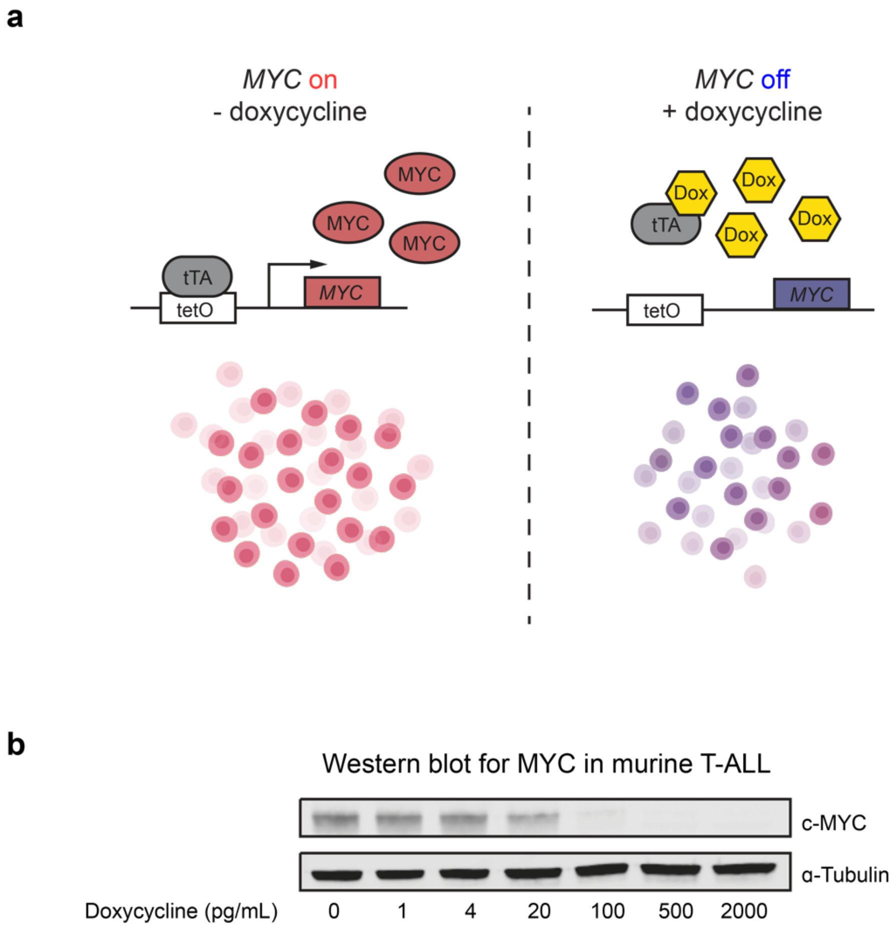

FIGURE S1: The *MYC* transgene is expressed in a tetracycline-off system

(a) Schematic for expression of the human *MYC* transgene under control of a tetracycline transactivator (tTA) in the murine EμSRα-*tTA*/tet-O-*MYC* model of T-ALL. Doxycycline binds to the tTA to prevent *MYC* expression.

(b) Western blot for MYC protein in murine T-ALL expressing the human *MYC* transgene 48 hours after addition of the indicated concentration of doxycycline. Data representative of two independent experiments.

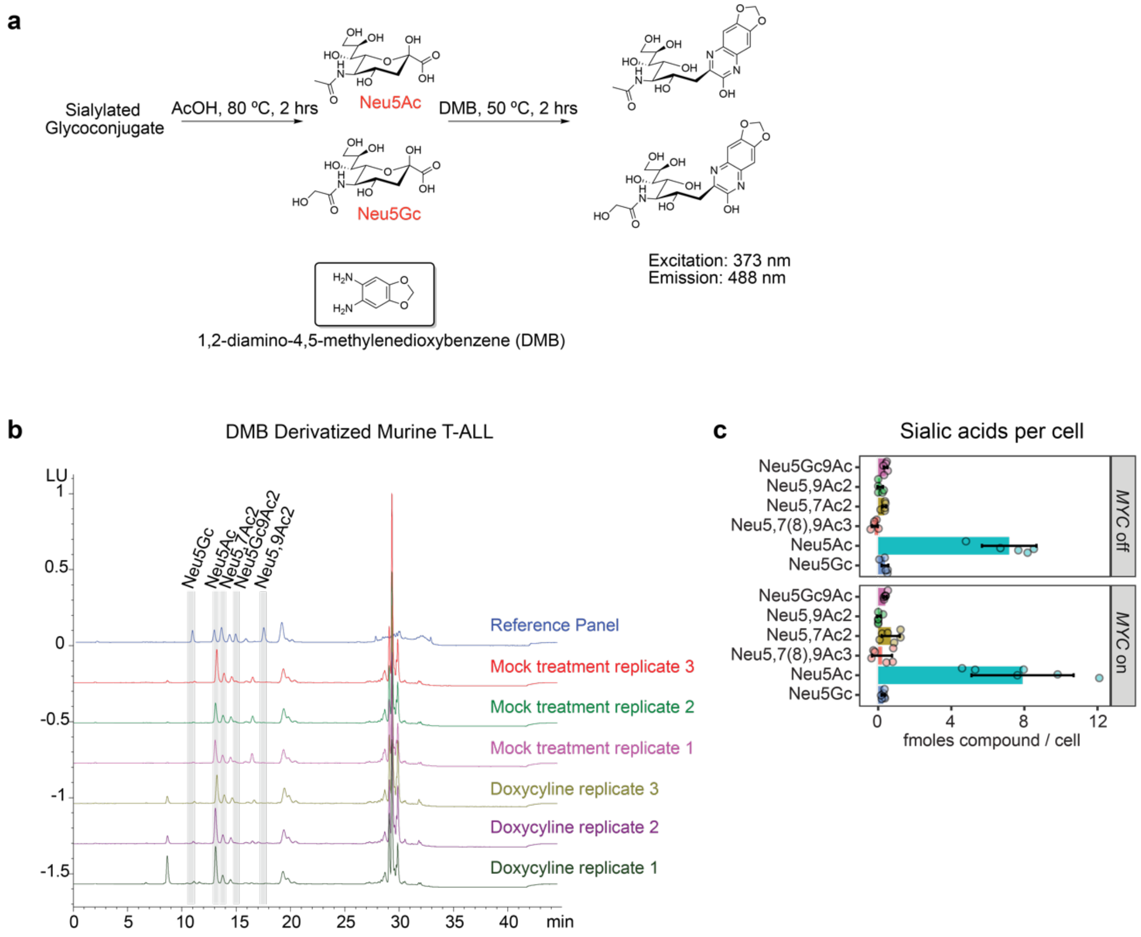

FIGURE S2: Concentrations of sialic acids in whole cell lysates are similar in *MYC* on/off states

(a) Assay for detection of sialic acids in whole cell lysate. Cell lysate is heated in 2M acetic acid at 80 ºC for 2 hours to release sialic acid from sialylated glycoconjugates. Free sialic acids are then labeled with 1,2-diamino-4,5-methylenedioxybenzene (DMB), separated by HPLC, and quantified by the fluorescence signal at 488 nm.

(b) Raw HPLC traces of a sialic acid reference panel, mock treated murine T-ALL (*MYC* on), and T-ALL treated with 500 pg/mL doxycycline (*MYC* off) for 48 hours. Peaks representing various sialic acids were assigned by alignment to the reference panel and are highlighted.

(c) Quantification of sialic acids per cell in murine T-ALL in the *MYC* on and off states (n=6 per sample, representative of two independent experiments). Data are mean ± s.d.

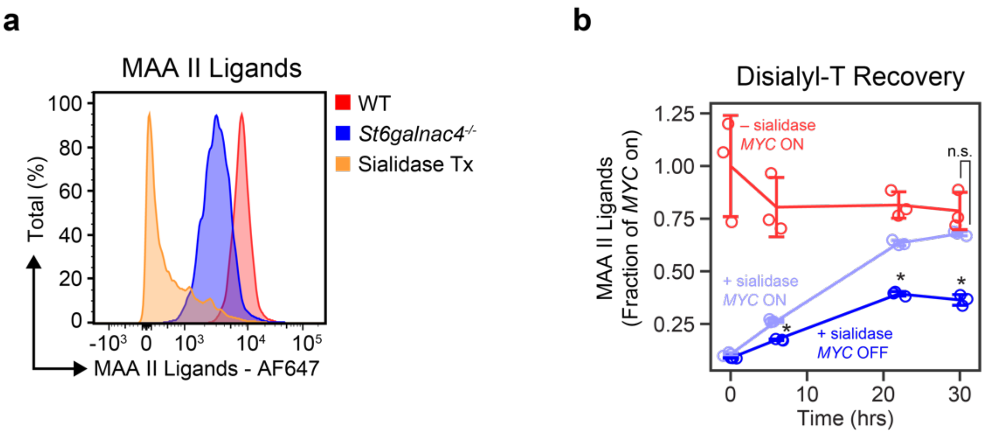

FIGURE S3: Kinetics of MAA II Ligand Recovery

(a) MAA II ligands decorating WT, *St6galnac4*$^{-/-}$, and *Vibrio cholerae* (VC) sialidase treated WT T-ALL cells.

(b) T-ALL was put in the *MYC* on or off state and then treated with VC sialidase to remove cell surface sialic acids. Recovery of MAA II ligands on the cell surface was monitored by flow cytometry over 30 hours (n=3 per group, two-tailed Student's *t* test comparing sialidase treated *MYC* on versus off cells at each time point, *$p<0.05$, data are representative of two independent experiments). Data are mean ± s.d.

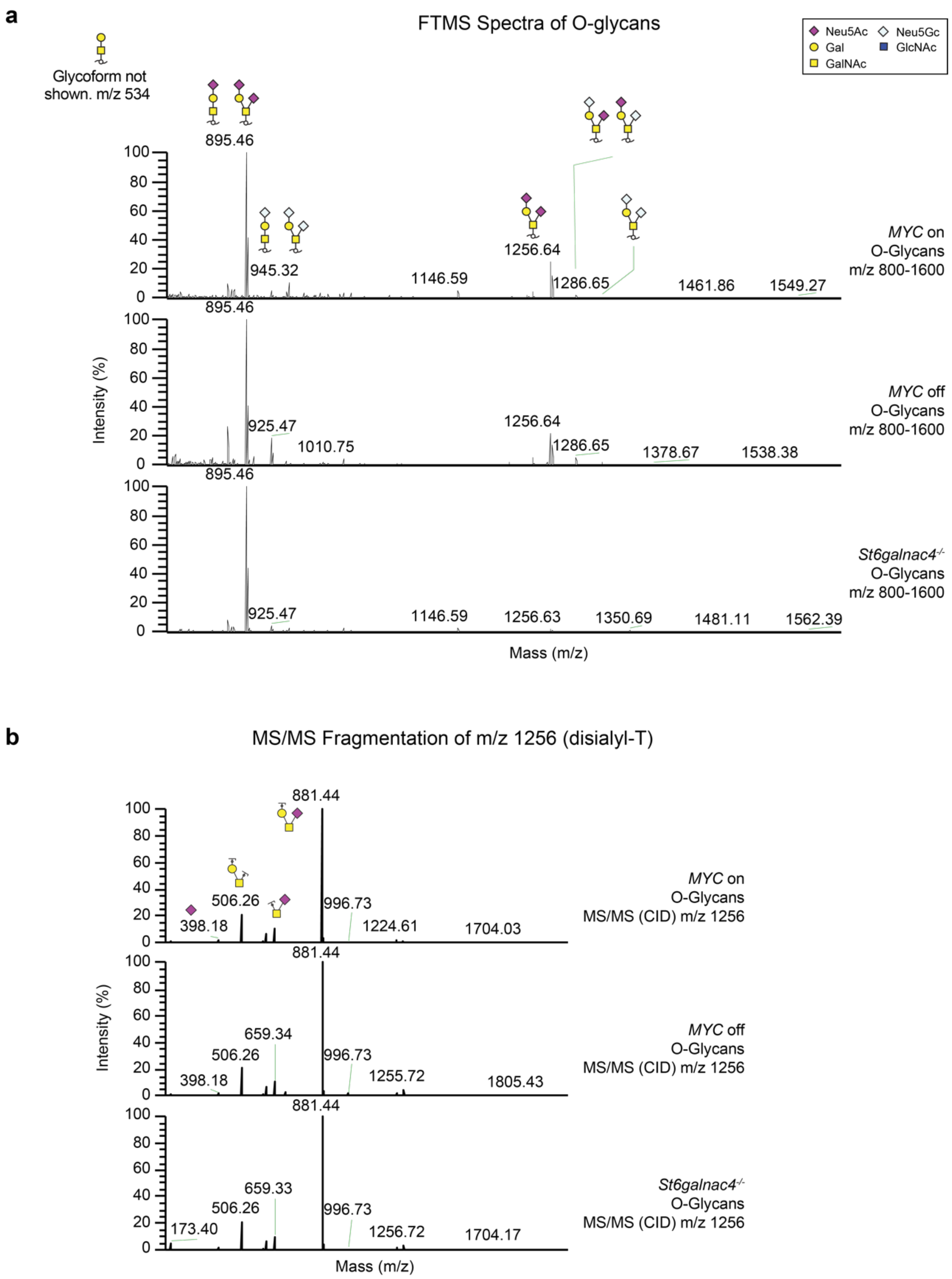

FIGURE S4: O-glycomics profiling by NSI-FTMS/MS

(a) Nanospray Ionization-Fourier Transform Mass Spectrometry (NSI-FTMS/MS) of murine T-ALL O-glycans in the *MYC* on and off states, as well as the corresponding *St6galnac4*$^{-/-}$ line.

(b) Spectra from MS/MS fragmentation of the peak at m/z of 1256, corresponding to disialyl-T, for each of the panels in **(a)**.

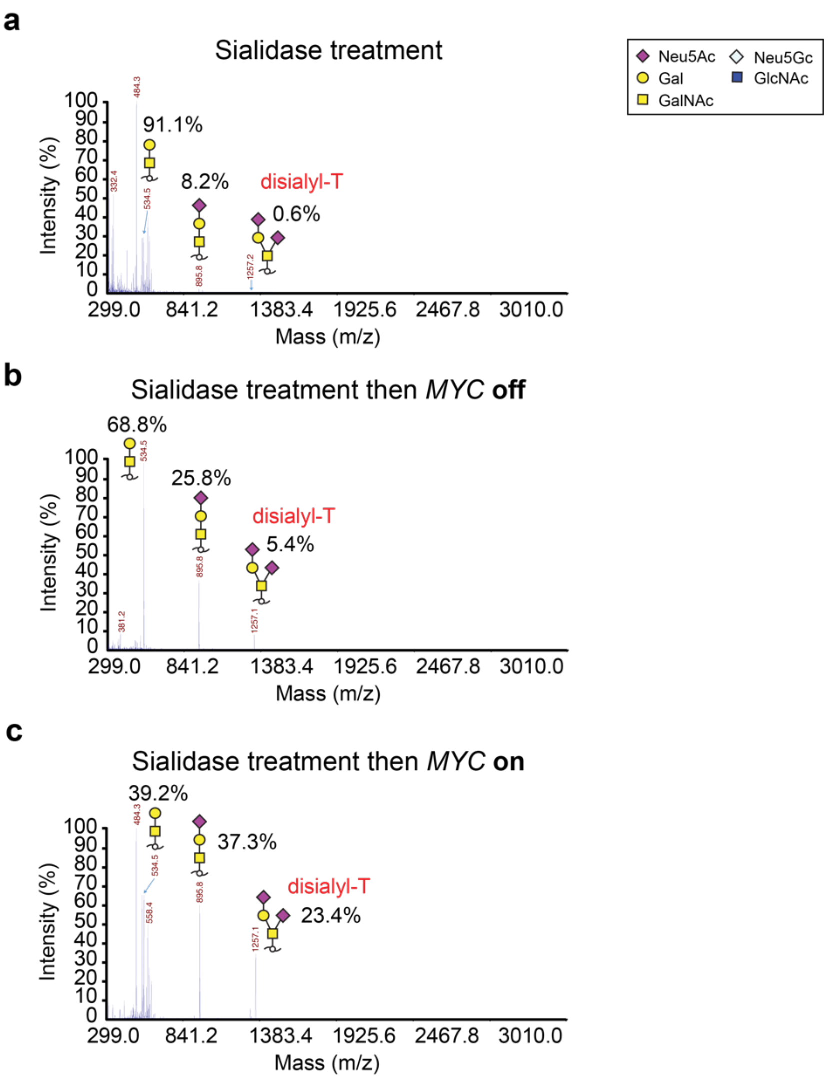

FIGURE S5: Recovery of disialyl-T after sialidase treatment

(a) O-glycans were profiled on T-ALL treated with VC sialidase by Matrix Assisted Laser Desorption Ionization-Time of Flight-Mass Spectrometry (MALDI-TOF-MS) following β-elimination.

(b,c) After treatment with sialidase as above, O-glycans on T-ALL in the *MYC* off **(b)** and on **(c)** states were allowed to recover for 48 hours prior to characterization by MALDI-TOF-MS.

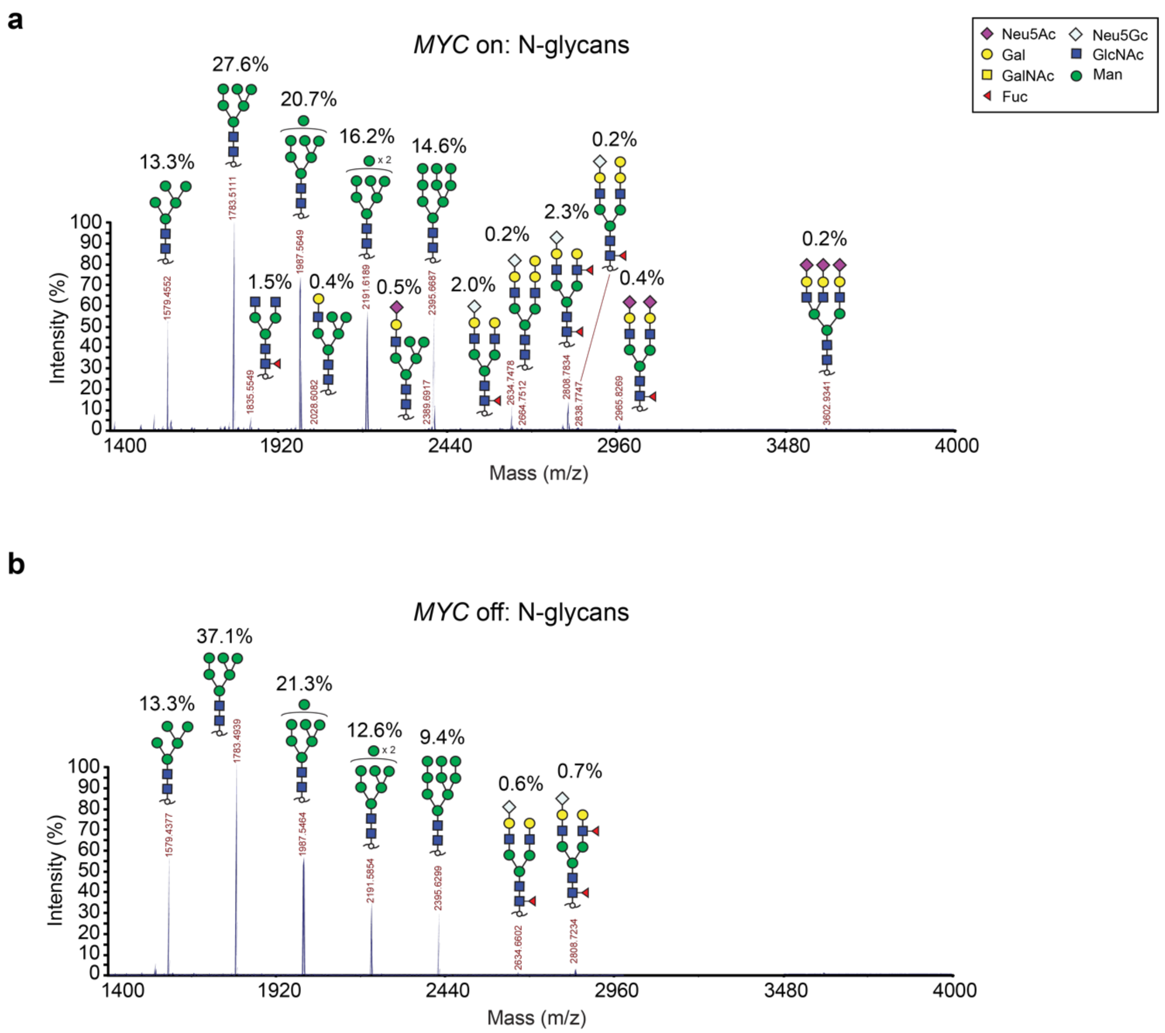

FIGURE S6: N-glycomics profiling by MALDI-TOF-MS

(a,b) Matrix Assisted Laser Desorption Ionization-Time of Flight-Mass Spectrometry (MALDI-TOF-MS) of murine T-ALL N-glycans in the *MYC* on **(a)** and off **(b)** states. The relative percent abundance of each glycan species in the sample is indicated.

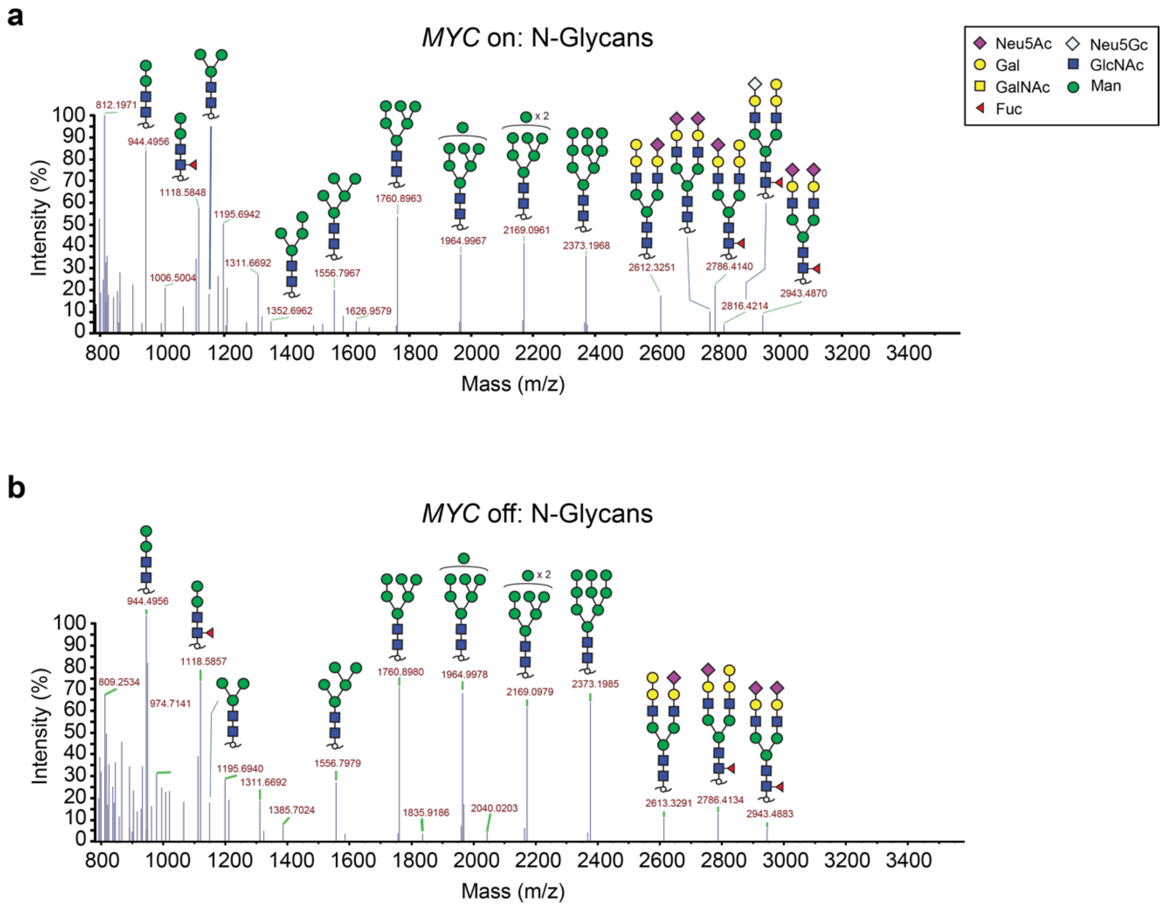

FIGURE S7: N-glycomics profiling by NSI-FTMS/MS

(a,b) Nanospray Ionization-Fourier Transform Mass Spectrometry (NSI-FTMS/MS) of murine T-ALL N-glycans in the MYC on **(a)** and off **(b)** states. Glycan structures were assigned to each mass by additional MS/MS fragmentation and comparison to a database.

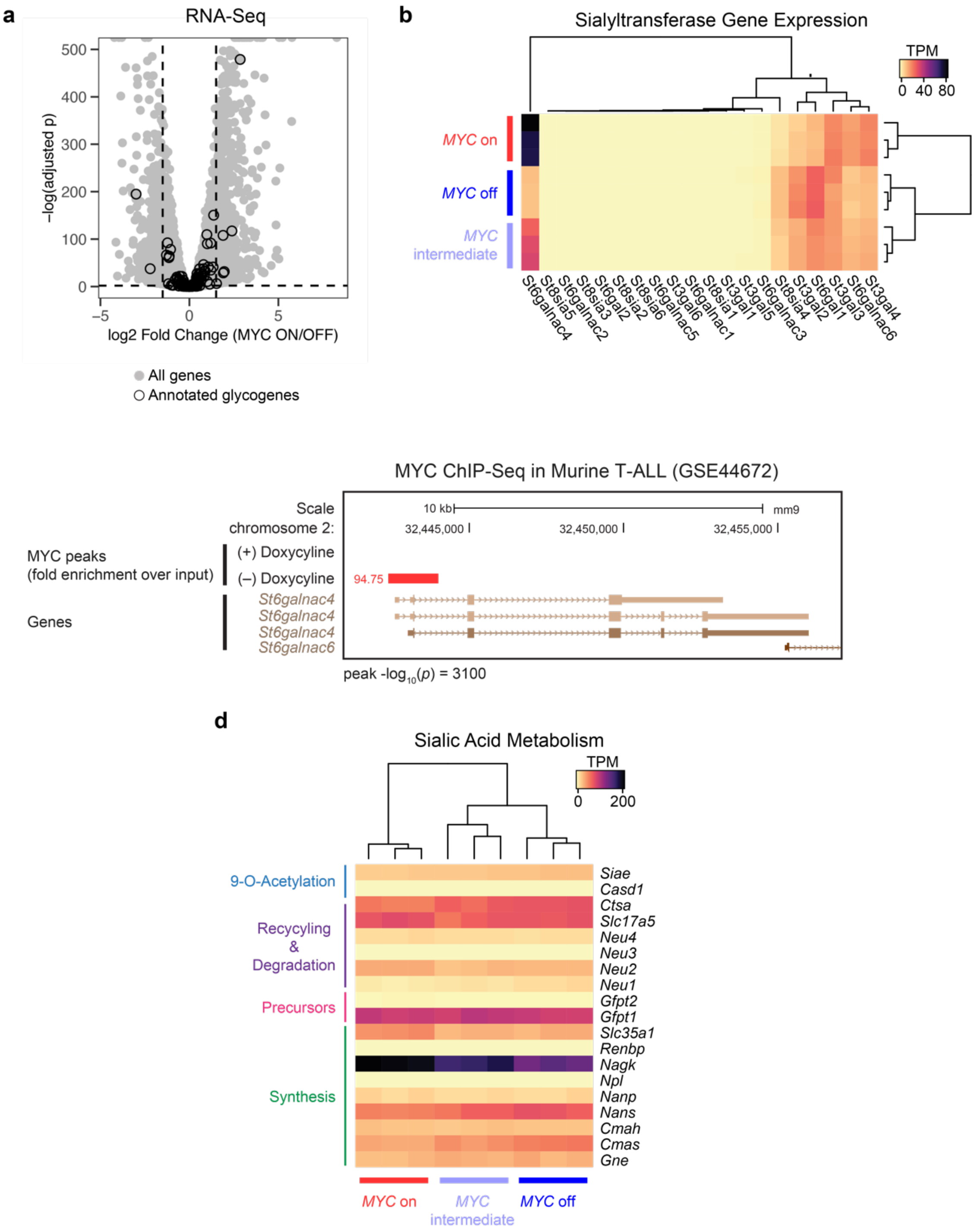

FIGURE S8: RNA-seq identifies changes in sialyltransferase expression between the *MYC* on/off states

(a) Volcano plot of differences in gene expression between murine T-ALL treated with 0 pg/mL doxycycline (*MYC* on) or 500 pg/mL doxycycline (*MYC* off) for 48 hours. Genes annotated to be involved in glycosylation are circled. Vertical dashed lines indicate a $\log_2$ fold change of 1.5.

(b) Heatmap of sialyltransferase gene expression across *MYC* on (0 pg/mL doxycycline), *MYC* off (500 pg/mL doxycycline), and *MYC* intermediate (20 pg/mL doxycycline) murine T-ALL

(n=3 per group). TPM, transcripts per million.

(c) Chromatin Immunoprecipitation-Sequencing (ChIP-Seq) for MYC in GSE44672(33), a dataset which analyzed the same murine T-ALL cells as in the present study. The fold-enrichment of the MYC peak relative to input is displayed for the *MYC* on (–Doxycycline) and *MYC* off (+Doxycycline) samples. Three isoforms of *St6galnac4* are shown. Graph produced using the UCSC Genome Browser (http://genome.ucsc.edu). Model-based analysis of ChIP-seq (MACS) was used to assign a *p*-value to detection of the peak.

(d) Heatmap of genes involved in sialic acid metabolism across *MYC* transgene states in murine T-ALL (n=3 per group). The function for each gene in sialic acid metabolism is indicated. TPM, transcripts per million.

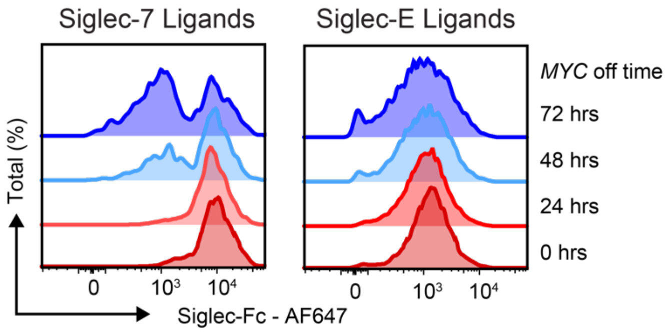

FIGURE S9: *MYC* supports display of Siglec-E/-7 ligands
Representative flow cytometry plots of ligands for Siglec-7 and Siglec-E on murine T-ALL at various times after turning *MYC* off. Data representative of three independent experiments.

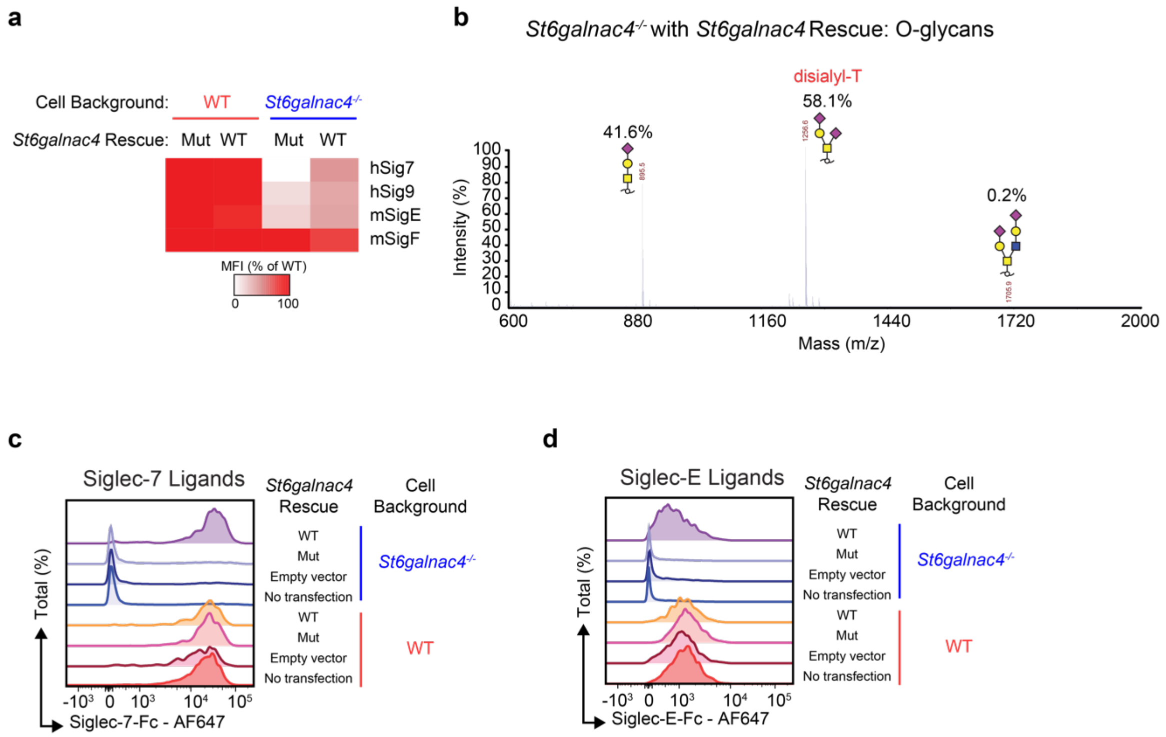

FIGURE S10: Transfection with *St6galnac4* rescues Siglec ligand display

(a) Heatmap of Siglec ligands displayed by control or *St6galnac4*$^{-/-}$ T-ALL rescued by transfection with a plasmid containing dead mutant or WT *St6galnac4*, as measured by flow cytometry following staining with the indicated Siglec-Fc reagent. Data are row normalized to control cells transfected with dead mutant *St6galnac4*. Plot is representative of two independent experiments. MFI, mean fluorescence intensity.

(b) O-glycan profiling by MALDI-TOF-MS of *St6galnac4*$^{-/-}$ T-ALL reexpressing *St6galnac4.*

(c,d) WT and *St6galnac4*$^{-/-}$ knockout murine T-ALL were transfected with either empty vector, mutant *St6galnac4*, or WT *St6galnac4* prior to staining for Siglec-7 **(c)** and Siglec-E **(d)** ligands. Plots are representative of three independent experiments.

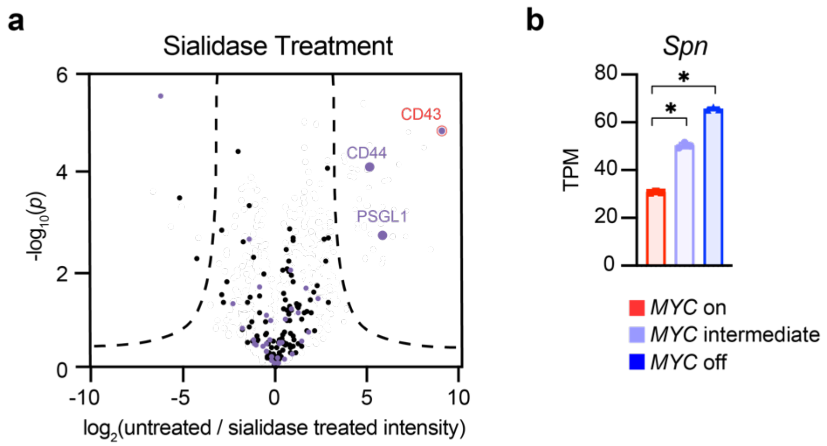

FIGURE S11: Siglec-7 immunoprecipitation mass spectrometry

(a) Proteins enriched by immunoprecipitation of T-ALL lysate with Siglec-7-Fc were identified by shotgun proteomics. Annotated cell surface or secreted proteins are displayed on the plot in black, with purple denoting the subset of glycoproteins. The intensity of spectra from untreated relative to VC sialidase-treated T-ALL is displayed (n=3 per group, significance cutoff by Student's *t* test with a false discovery rate of 0.0001 and minimum enrichment (S_0) of 5).

(b) Expression of *Spn*, the gene for CD43, by RNA-seq in T-ALL in the *MYC* on, intermediate, and off states (n=3 per group, two-tailed Student's *t* test, *p<0.0001).

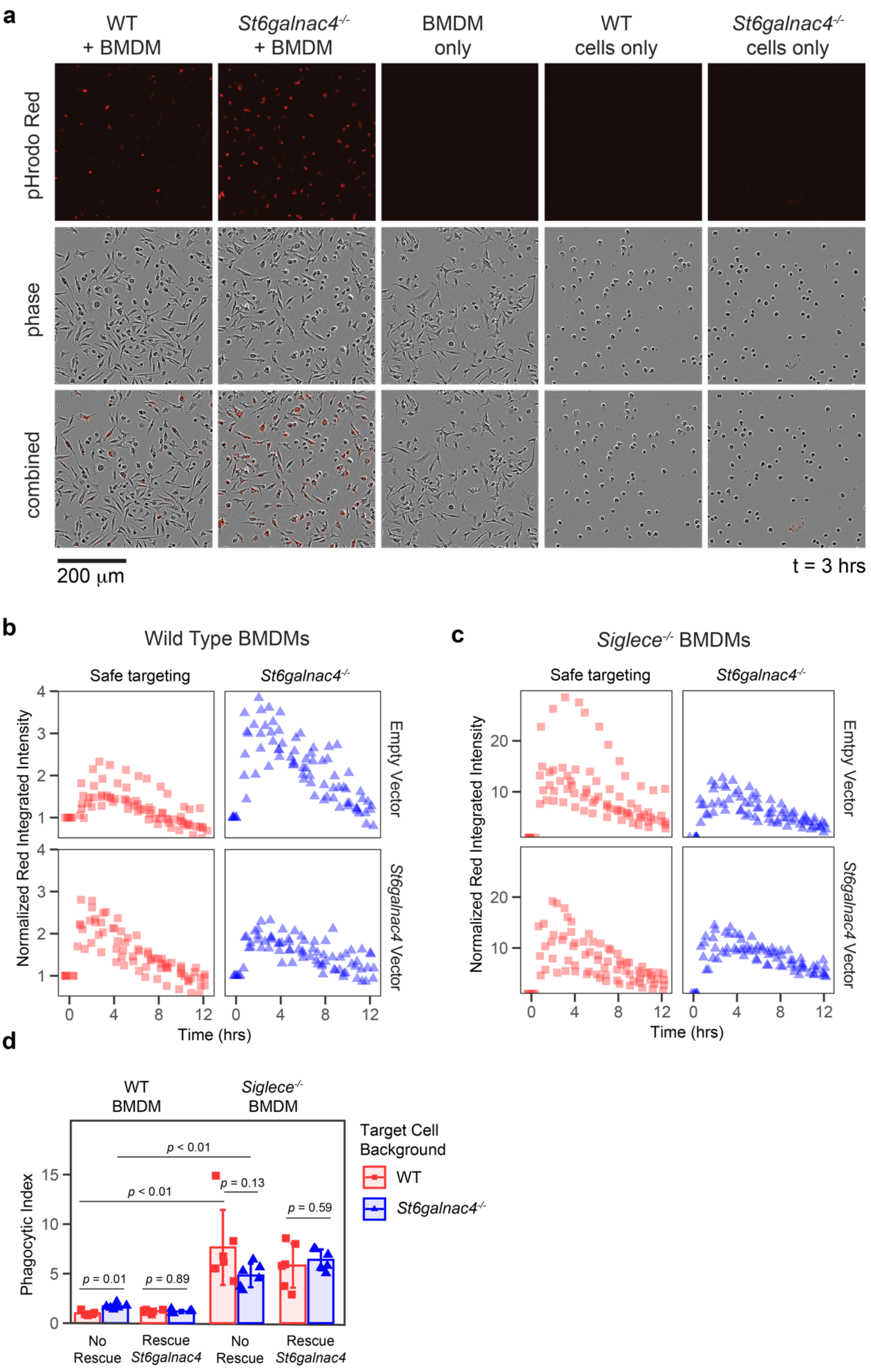
a
WT + BMDM
St6galnac4-/- + BMDM
BMDM only
WT cells only
St6galnac4-/- cells only
pHrodo Red
phase
combined
200 μm
t = 3 hrs
b
Wild Type BMDMs
Safe targeting
St6galnac4-/-
Empty Vector
St6galnac4 Vector
Normalized Red Integrated Intensity
Time (hrs)
c
Siglece-/- BMDMs
Safe targeting
St6galnac4-/-
Empty Vector
St6galnac4 Vector
Normalized Red Integrated Intensity
Time (hrs)
d
WT BMDM
Siglece-/- BMDM
Phagocytic Index
p < 0.01
p < 0.01
p = 0.13
p = 0.59
p = 0.01
p = 0.89
No Rescue
Rescue St6galnac4
No Rescue
Rescue St6galnac4
Target Cell Background
WT
St6galnac4-/-

FIGURE S12: Live cell phagocytosis by bone marrow derived macrophages

(a) Representative images of phagocytosis of WT and *St6galnac4*$^{-/-}$ T-ALL labeled with pHrodo red by syngeneic bone marrow derived macrophages (BMDMs) at t = 3 hrs. Images are representative of three independent experiments.

(b,c) Time course of murine WT **(b)** and *Siglece*$^{-/-}$ **(c)** BMDMs phagocytosing the indicated murine T-ALL line over 12 hours. Fluorescence integrated intensities were normalized to empty vector transfected WT target cells.

(d) Side-by-side comparison of the plots in main **Fig. 2g** and **h** to compare phagocytosis by WT and *Siglece*$^{-/-}$ BMDMs (n=6 per group, two-tailed Student's t test).

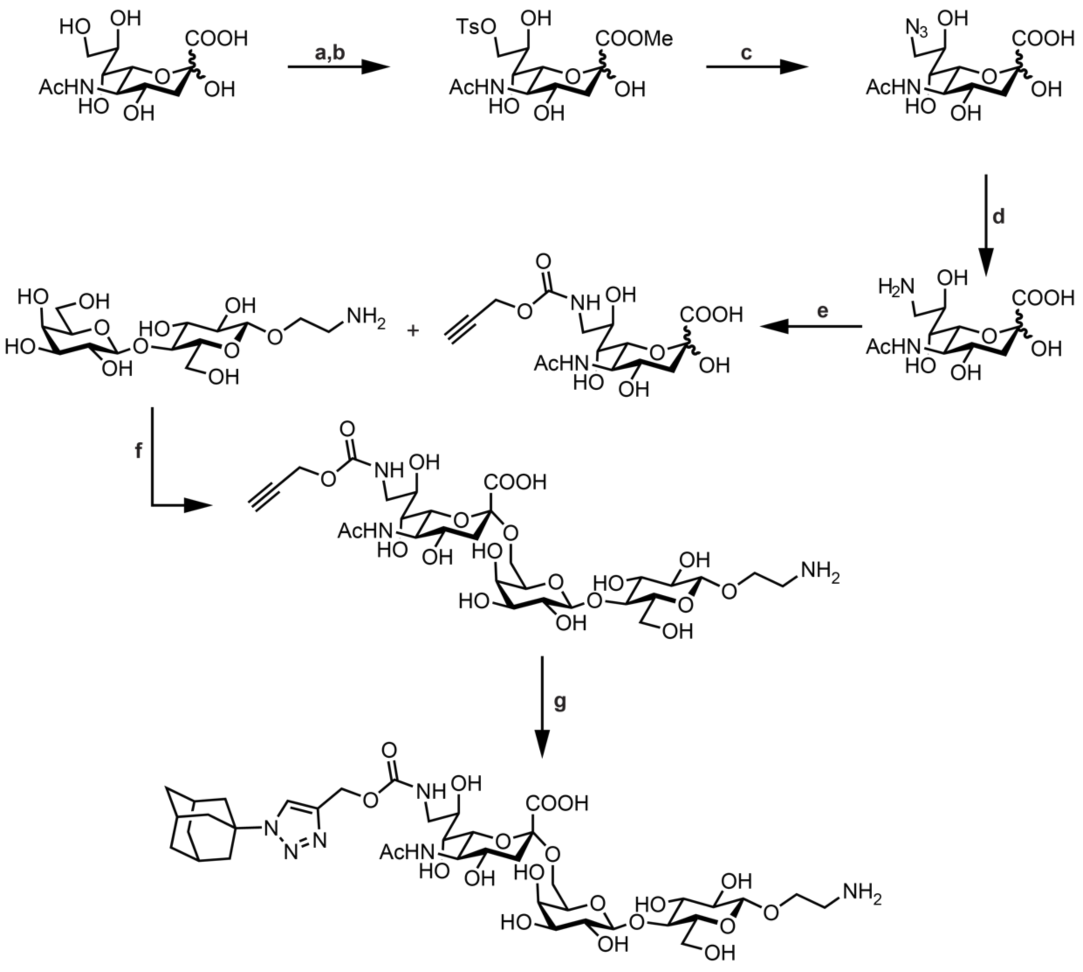

FIGURE S13: Synthesis of Siglec-E inhibitor

(a) Amberlite-120 H^+ resin, methanol, 20 °C, 48 h.

(b) p-Toluenesulfonylchloride, pyridine, 20 °C, 24 h; 41% over two steps.

(c) Sodium azide, 3:1 acetone to water, 80 °C, 16 h; 74%.

(d) Pearlman's catalyst, $H_{2(g)}$, methanol, 20 °C, 2 h; 100%.

(e) Propargylchloroformate, Hünig's base, methanol, 20 °C, 24 h; 12%.

(f) Cytidine triphosphate, pH 7.4 100 mM Tris with 20 mM $MgCl_2$, Pd26ST, NmCSS, 37 °C, 4 h; 13%.

(g) Azido adamantane, sodium ascorbate, BTTAA, $CuSO_4$, 1:1 water/tert-butanol, 20 °C, 48 h; 53%.

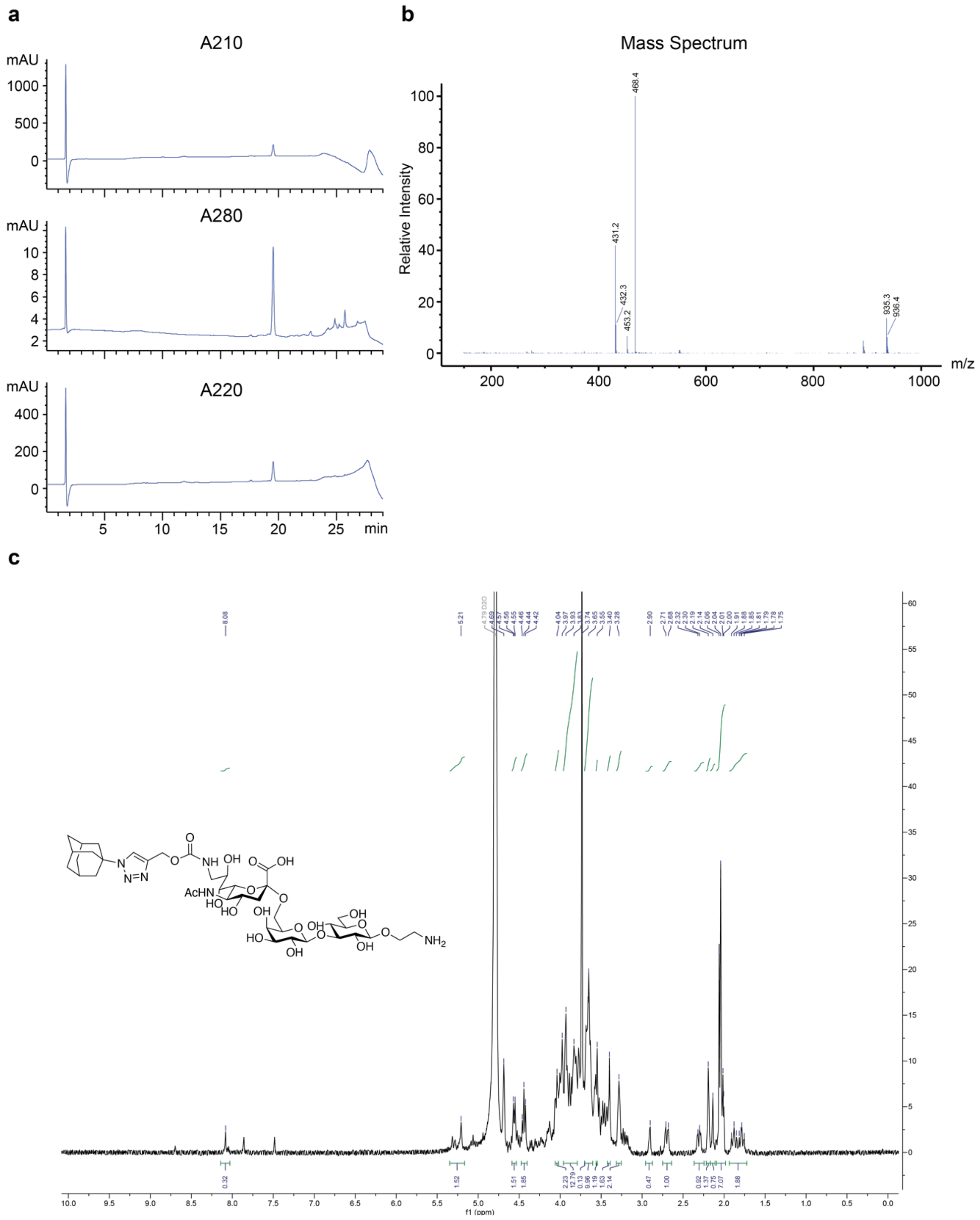

FIGURE S14: Siglec-E inhibitor analytical data

(a,b) Liquid chromatography mass spectrometry traces (LC/MS) of the Siglec-E inhibitor showing absorbance at 210, 220, and 280 nm **(a)** and mass spectrum of the main peak acquired by electrospray ionization, revealing the $[M+H]^+$ peak at 935.3 **(b)**.

(c) 1H NMR trace of the Siglec-E inhibitor.

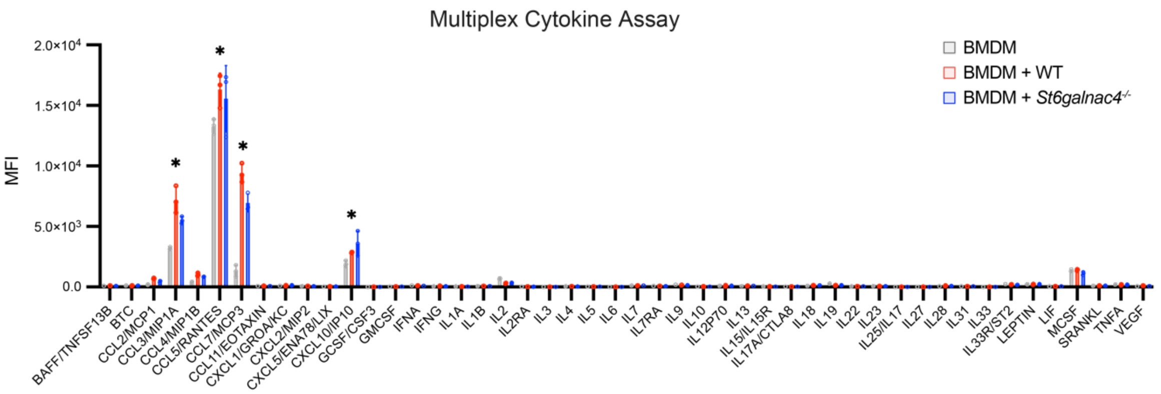

FIGURE S15: Multiplex cytokine panel

Multiplex cytokine panel of BMDMs incubated with anti-Thy1.1 antibody plus WT or *St6galnac4*$^{-/-}$ target cells (n=3 per group, two-way ANOVA using a single family and Tukey's test for multiple comparisons, * is a significant change from control with adjusted *p*<0.0001). See main Fig. 2l for close-up.

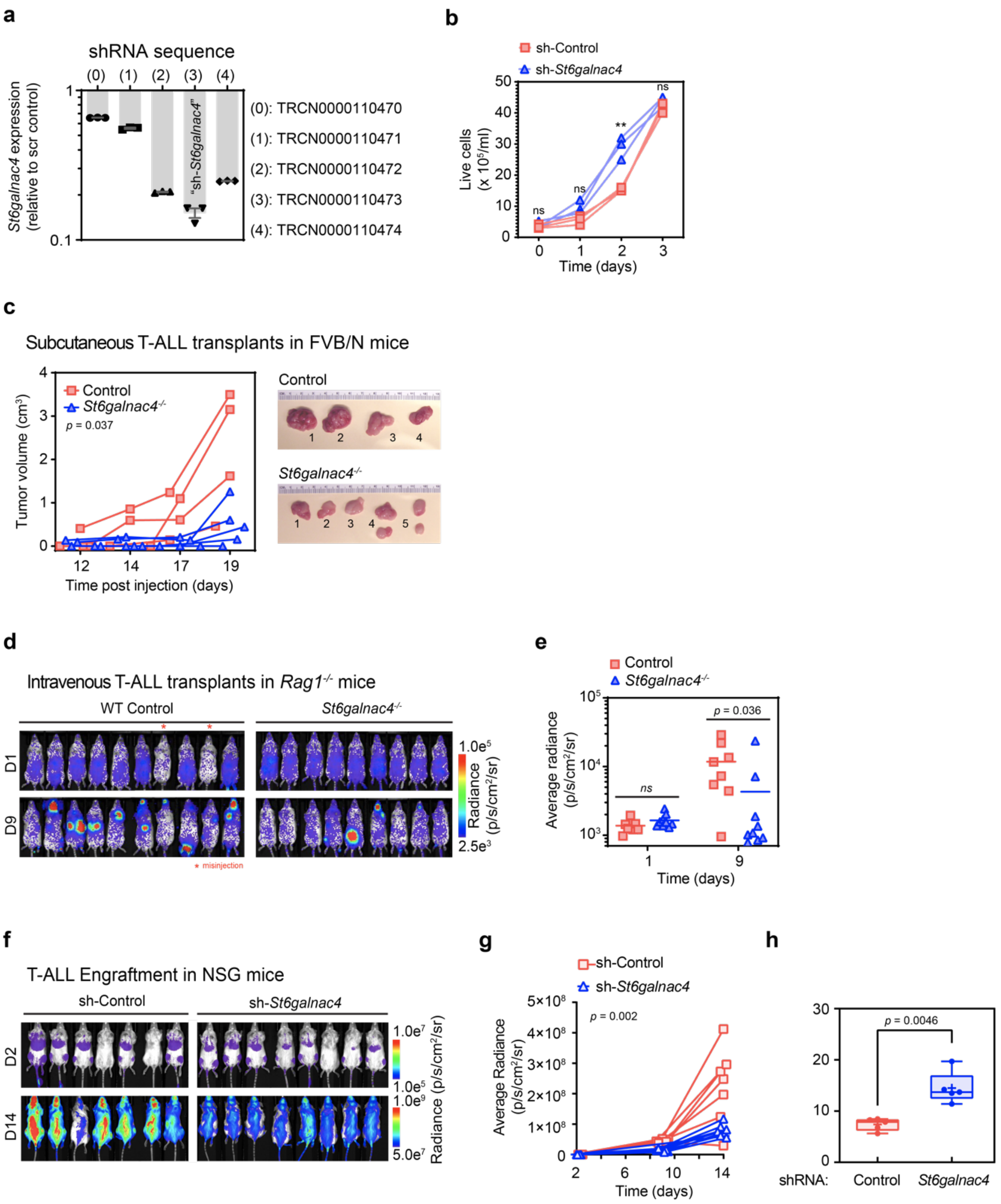

FIGURE S16: T-ALL growth *in vivo* is promoted by *St6galnac4*

(a) Quantitative RT-PCR of *St6galnac4* expression in MYC-driven T-ALL cells upon down-regulation using 5 different *St6galnac4*-specific shRNAs and a scrambled control.

(b) *In vitro* cell proliferation of MYC-driven T-ALL cells expressing either a scrambled control or a *St6galnac4*-specific shRNA (n=3, Student's *t* test, ns: not significant, ***p* = 0.0029).

(c) Subcutaneous transplants of MYC-driven WT (safe targeting) or *St6galnac4*$^{-/-}$ T-ALL in syngeneic FVB/N mice. Tumor volume was assessed by caliper measurement [n(Control)=4, n(*St6galnac4*$^{-/-}$)=5, two-way ANOVA]. Gross tumors at time of euthanasia are shown.

(d) Bioluminescence images of IV transplants of luciferase-labeled MYC-driven WT or

St6galnac4$^{-/-}$ T-ALL in syngeneic *Rag1*$^{-/-}$ mice. Images show T-ALL burden one day (D1) and nine days (D9) post injection.

(e) Tumor burden of mice described in (d) was determined based on bioluminescence average radiance (left). Relative tumor growth was assessed by $\log_2$-fold change of average radiance (right). n(Control)=8, n(*St6galnac4*$^{-/-}$)=9, Mann-Whitney *U* test, ns: not significant.

(f) Bioluminescence imaging of NSG mice transplanted IV with luciferase-labeled MYC-driven T-ALL cells expressing either a *St6galnac4*-specific or a control shRNA. Images show tumor burden on day two and fourteen post transplantation.

(g) Tumor growth was assessed by bioluminescence imaging over time and quantified [n(Control)=7, n(sh-*St6galnac4*$^{-/-}$)=8, Mixed Effects analysis].

(h) Flow cytometric analysis of splenocytes isolated from NSG mice described in (f), 14 days after IV-transplantation of MYC-driven T-ALL expressing either an *St6galnac4*-specific or a control shRNA. Frequency of CD11b-positive cells was compared between the two groups (n=5 mice per group, two-tailed Welch's *t* test).

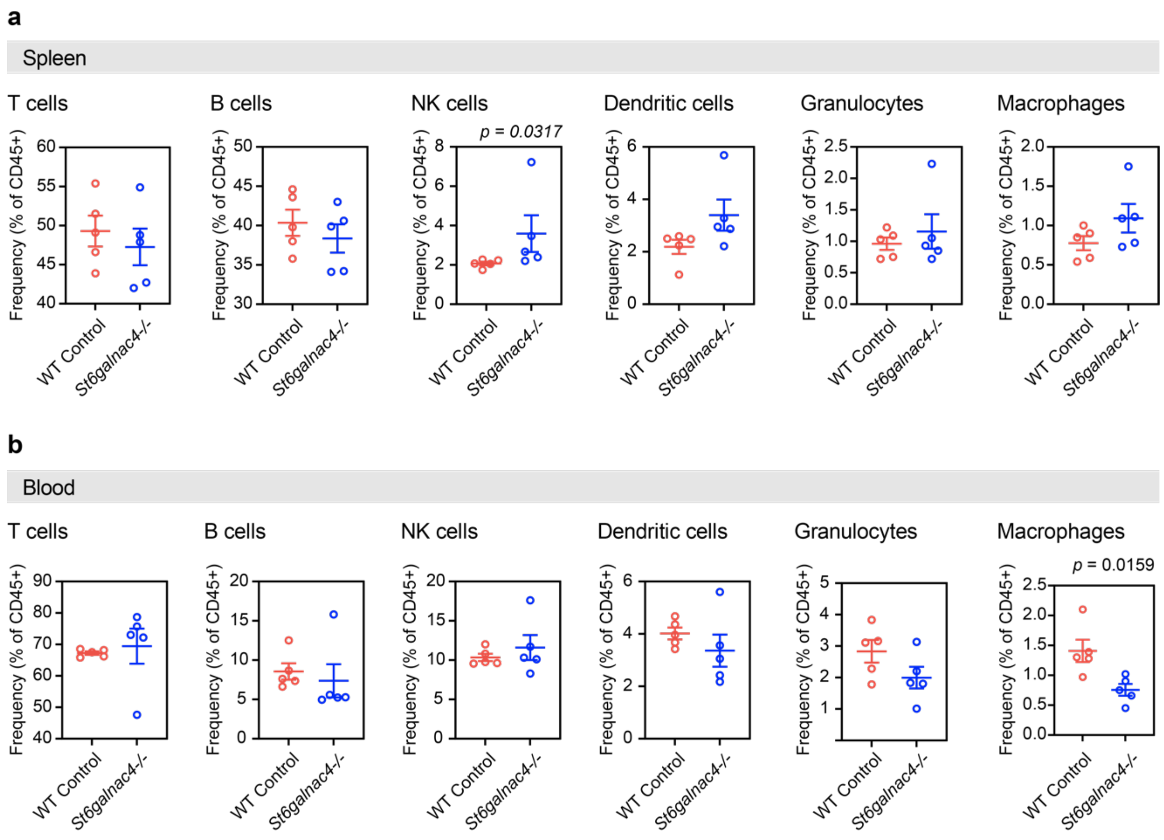

FIGURE S17: T-ALL *St6galnac4* expression affects immune status *in vivo*

(a,b) Flow cytometric analysis of splenocytes (a) and peripheral blood mononuclear cells (b) isolated from FVB/N mice 23 days after IV-transplantation of MYC-driven T-ALL expressing either wild type *St6galnac4* (WT Control, n= 5) or lacking *St6galnac4* expression (*St6galnac4*$^{-/-}$, n= 5). Frequencies of indicated immune cell subsets are shown per mouse (two-tailed Mann-Whitney test).

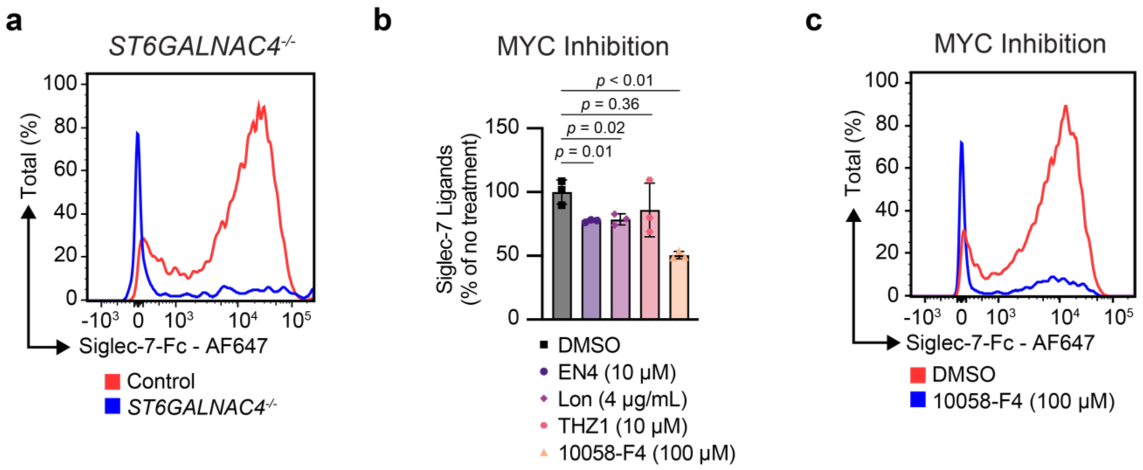

FIGURE S18: Siglec-7 ligand inhibition in human T-ALL

(a) Representative plot of Siglec-7 ligands on safe targeting control and *ST6GALNAC4*$^{-/-}$ human T-ALL (PEER) cells.

(b) Staining for Siglec-7 ligands on PEER cells following treatment with various MYC inhibitors for 48 hours at the indicated concentration (n=3 per group, two-tailed Student's *t* test). Data presented as mean ± s.d.

(c) Representative plot of Siglec-7 ligands on PEER cells after treatment with DMSO or 100 μM 10058-F4 for 48 hours.

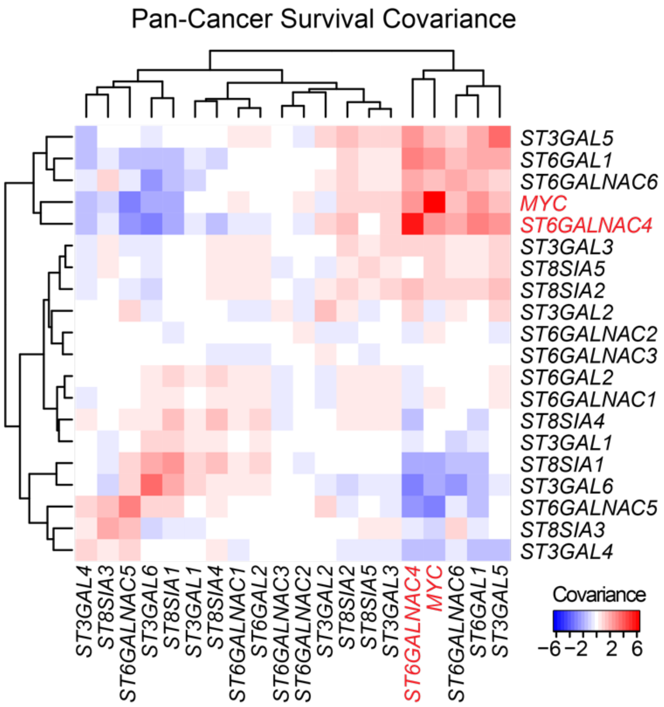

FIGURE S19: Pan-cancer survival covariance
Survival Z-scores for all cancers in PRECOG(24) were extracted after stratifying patients by median expression of the indicated gene. A covariance matrix was then computed using the twenty sialyltransferases and *MYC*. A high positive covariance (red) indicates that two genes have similar effects on patient survival.

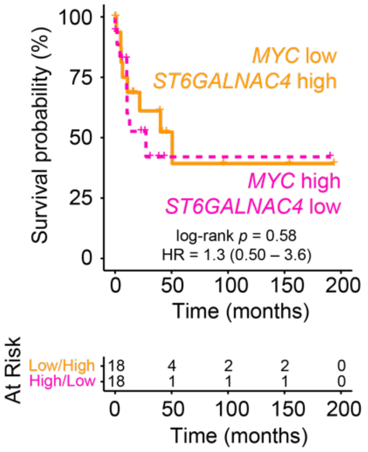

FIGURE S20: Survival in diffuse large B cell lymphoma
Overall survival in a cohort of DLBCL patients that do not have the signature of malignant glycosylation defined as high *MYC* and high *ST6GALNAC4*, stratified by median expression level (GSE4475)(25).

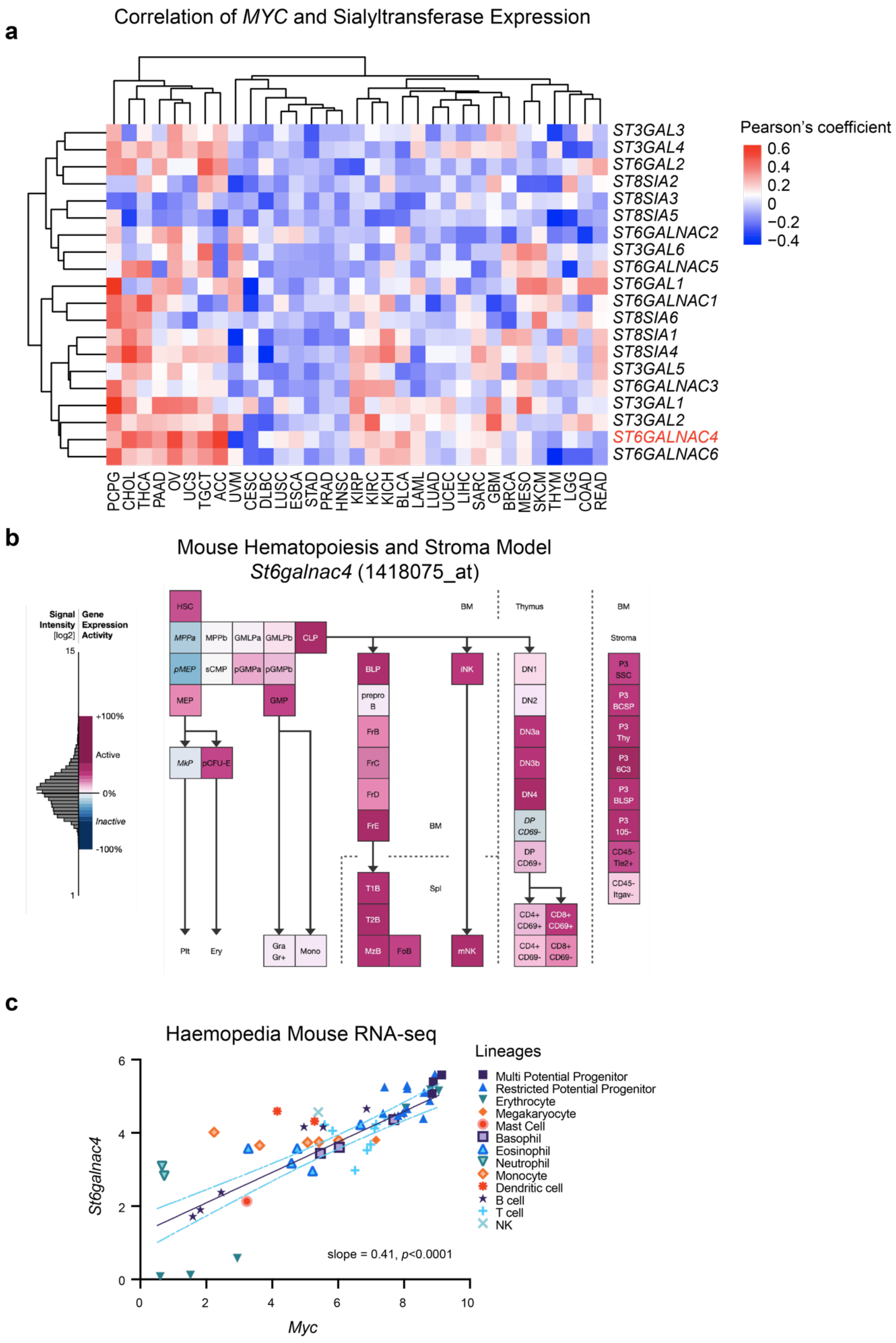

FIGURE S21: *MYC* and *ST6GALNAC4* across tissues and in hematopoiesis
(a) Heatmap of Pearson's coefficients for correlation between *MYC* and each of the twenty

sialyltransferases based on mRNA expression using TCGA datasets (positive correlation: red, no correlation: white, negative correlation: blue).

(b) Expression of *St6galnac4* during murine hematopoiesis, normalized and displayed by Gene Expression Commons (https://gexc.riken.jp/models/1507/genes/St6galnac4)(23, 34).

(c) Correlation of *Myc* and *St6galnac4* expression in various murine hematopoietic lineages from Haemopedia(35).

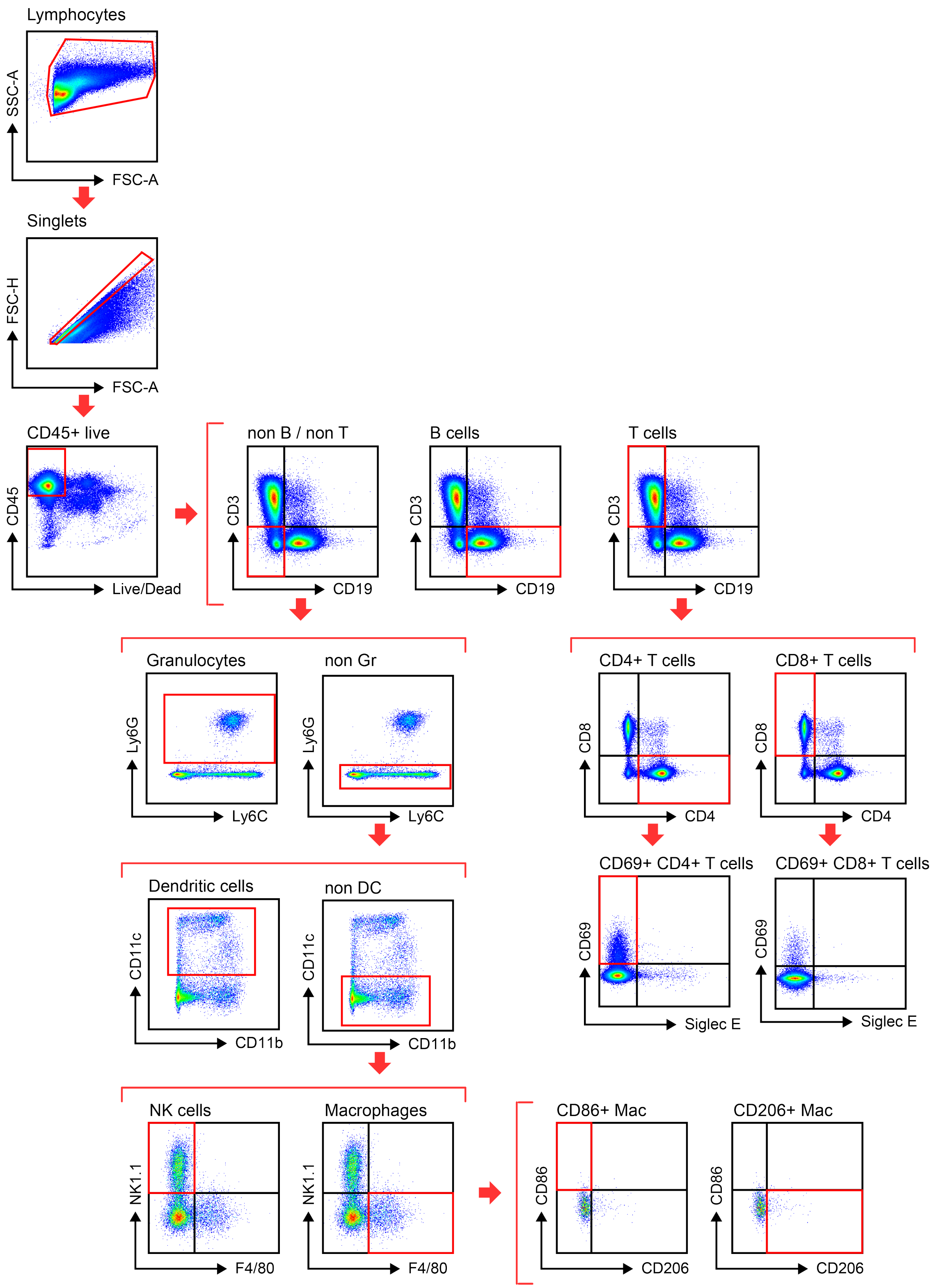

FIGURE S22: Gating strategy for flow cytometric phenotyping of T-ALL bearing FVB/N mice

Gating strategy for flow cytometry-based frequency analysis of major immune subsets, T cell activation, and macrophage polarization in FVB/N mice bearing syngeneic MYC-driven T-ALL.

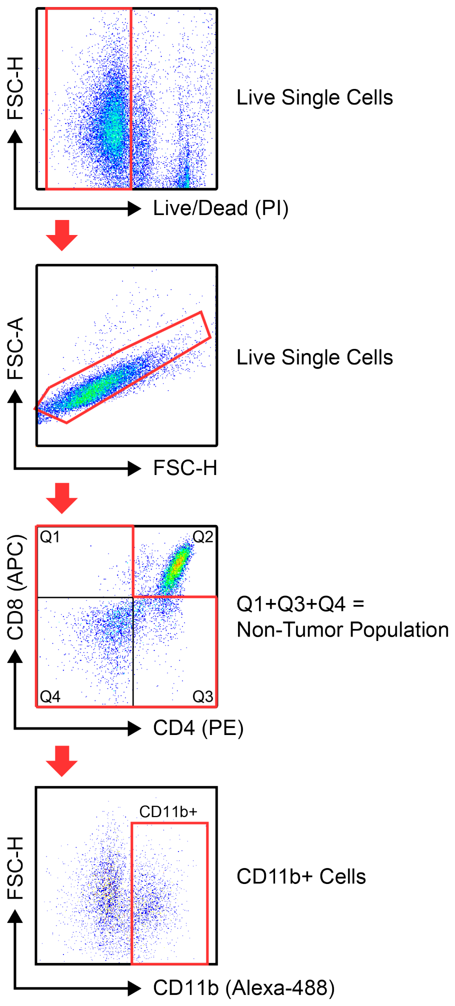

FIGURE S23: Gating strategy for flow cytometric analysis of T-ALL bearing NSG mice
Gating strategy for flow cytometry-based analysis of tumors in NSG mice.

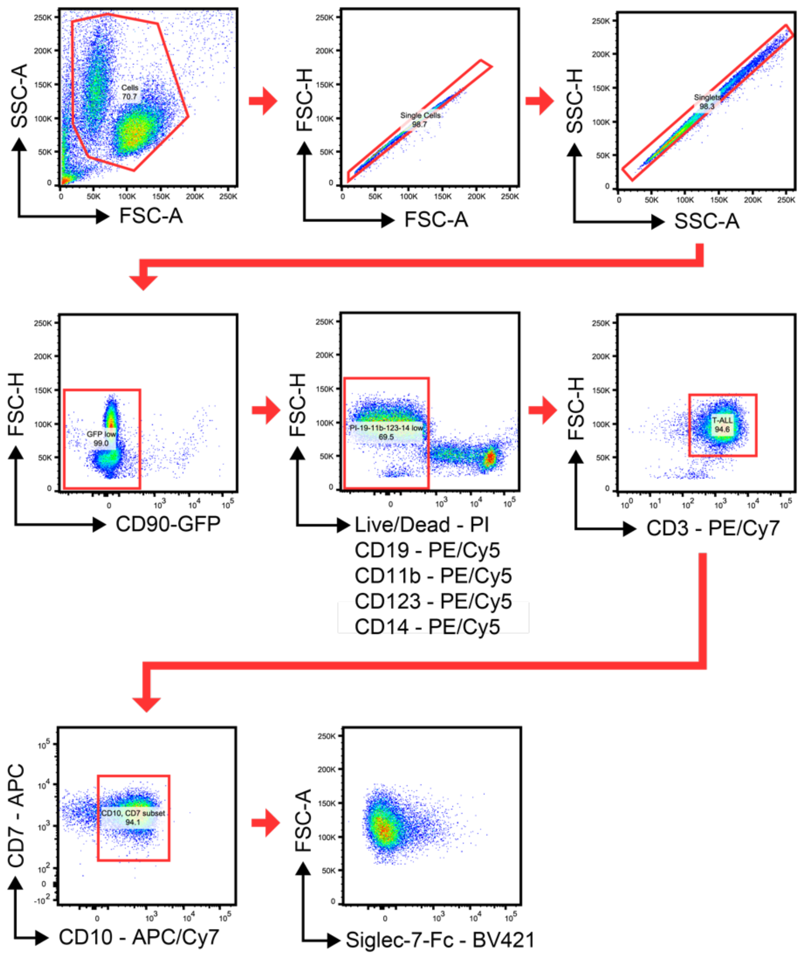

FIGURE S24: Gating strategy for flow cytometric analysis of human T-ALL samples
Gating strategy for flow cytometry-based sorting and analysis of primary human T-ALL samples.

5

Supplementary Tables

Table S1. (separate file)
Processed RNA-seq data comparing MYC dose in T-ALL.

Table S2. (separate file)
GO term analysis of *MYC* on and off T-ALL.

Table S3. (separate file)
List of glycogenes.

Table S4. (separate file)
Processed RNA-seq data of *MYC* off time course in T-ALL.

Table S5. (separate file)
Shotgun proteomics of Siglec-7 immunoprecipitation of T-ALL.

Table S6. (separate file)
Collated expression of *MYC* and *ST6GALNAC4* in human T-ALL.

SI References

1. R. C. Team, Others, R: A language and environment for statistical computing (2013).
2. H. Wickham, *et al.*, Welcome to the Tidyverse. *Journal of Open Source Software* **4**, 1686 (2019).
3. Y. Li, A. Deutzmann, P. S. Choi, A. C. Fan, D. W. Felsher, BIM mediates oncogene inactivation-induced apoptosis in multiple transgenic mouse models of acute lymphoblastic leukemia. *Oncotarget* **7**, 26926–26934 (2016).
4. N. E. Sanjana, O. Shalem, F. Zhang, Improved vectors and genome-wide libraries for CRISPR screening. *Nat. Methods* **11**, 783–784 (2014).
5. E. Kowarz, D. Löscher, R. Marschalek, Optimized Sleeping Beauty transposons rapidly generate stable transgenic cell lines. *Biotechnol. J.* **10**, 647–653 (2015).
6. Y. Zeng, T. N. C. Ramya, A. Dirksen, P. E. Dawson, J. C. Paulson, High-efficiency labeling of sialylated glycoproteins on living cells. *Nature Methods* **6**, 207–209 (2009).
7. M. R. Bond, *et al.*, Metabolism of diazirine-modified N-acetylmannosamine analogues to photo-cross-linking sialosides. *Bioconjug. Chem.* **22**, 1811–1823 (2011).
8. A. Dobin, *et al.*, STAR: ultrafast universal RNA-seq aligner. *Bioinformatics* **29**, 15–21 (2013).
9. Y. Liao, G. K. Smyth, W. Shi, The R package Rsubread is easier, faster, cheaper and better for alignment and quantification of RNA sequencing reads. *Nucleic Acids Res.* **47**, e47 (2019).
10. M. I. Love, W. Huber, S. Anders, Moderated estimation of fold change and dispersion for RNA-seq data with DESeq2. *Genome Biol.* **15**, 550 (2014).
11. S. Durinck, P. T. Spellman, E. Birney, W. Huber, Mapping identifiers for the integration of genomic datasets with the R/Bioconductor package biomaRt. *Nat. Protoc.* **4**, 1184–1191 (2009).
12. A. Alexa, J. Rahnenfuhrer, topGO: enrichment analysis for gene ontology. *R package version* **2**, 2010 (2010).
13. H. Narimatsu, Construction of a human glycogene library and comprehensive functional analysis. *Glycoconj. J.* **21**, 17–24 (2004).
14. A. Sabò, *et al.*, Selective transcriptional regulation by Myc in cellular growth control and lymphomagenesis. *Nature* **511**, 488–492 (2014).
15. D. F. Liefwalker, *et al.*, Metabolic convergence on lipogenesis in RAS, BCR-ABL, and MYC-driven lymphoid malignancies. *Cancer Metab* **9**, 31 (2021).
16. S. Tyanova, T. Temu, J. Cox, The MaxQuant computational platform for mass spectrometry-based shotgun proteomics. *Nat. Protoc.* **11**, 2301–2319 (2016).
17. J. Cox, *et al.*, Andromeda: a peptide search engine integrated into the MaxQuant environment. *J. Proteome Res.* **10**, 1794–1805 (2011).
18. J. E. Elias, S. P. Gygi, Target-decoy search strategy for increased confidence in large-scale protein identifications by mass spectrometry. *Nat. Methods* **4**, 207–214 (2007).

19. S. Tyanova, *et al.*, The Perseus computational platform for comprehensive analysis of (prote)omics data. *Nat. Methods* **13**, 731–740 (2016).

20. M. M. Spain, *et al.*, The RSC complex localizes to coding sequences to regulate Pol II and histone occupancy. *Mol. Cell* **56**, 653–666 (2014).

21. H. G. LaBreche, J. R. Nevins, E. Huang, Integrating factor analysis and a transgenic mouse model to reveal a peripheral blood predictor of breast tumors. *BMC Med. Genomics* **4**, 61 (2011).

22. M. Shi, *et al.*, A blood-based three-gene signature for the non-invasive detection of early human hepatocellular carcinoma. *Eur. J. Cancer* **50**, 928–936 (2014).

23. J. Seita, *et al.*, Gene Expression Commons: an open platform for absolute gene expression profiling. *PLoS One* **7**, e40321 (2012).

24. A. J. Gentles, *et al.*, The prognostic landscape of genes and infiltrating immune cells across human cancers. *Nat. Med.* **21**, 938–945 (2015).

25. M. Hummel, *et al.*, A biologic definition of Burkitt's lymphoma from transcriptional and genomic profiling. *N. Engl. J. Med.* **354**, 2419–2430 (2006).

26. Z. Tang, *et al.*, GEPIA: a web server for cancer and normal gene expression profiling and interactive analyses. *Nucleic Acids Res.* **45**, W98–W102 (2017).

27. V. Thorsson, *et al.*, The Immune Landscape of Cancer. *Immunity* **48**, 812-830.e14 (2018).

28. J. Vivian, *et al.*, Toil enables reproducible, open source, big biomedical data analyses. *Nat. Biotechnol.* **35**, 314–316 (2017).

29. B. Li, C. N. Dewey, RSEM: accurate transcript quantification from RNA-Seq data with or without a reference genome. *BMC Bioinformatics* **12**, 323 (2011).

30. W. E. Johnson, C. Li, A. Rabinovic, Adjusting batch effects in microarray expression data using empirical Bayes methods. *Biostatistics* **8**, 118–127 (2007).

31. H. Yu, *et al.*, Highly efficient chemoenzymatic synthesis of naturally occurring and non-natural alpha-2,6-linked sialosides: a P. damsela alpha-2,6-sialyltransferase with extremely flexible donor-substrate specificity. *Angew. Chem. Int. Ed Engl.* **45**, 3938–3944 (2006).

32. C. D. Rillahan, E. Schwartz, R. McBride, V. V. Fokin, J. C. Paulson, Click and pick: identification of sialoside analogues for siglec-based cell targeting. *Angew. Chem. Int. Ed Engl.* **51**, 11014–11018 (2012).

33. S. Walz, *et al.*, Activation and repression by oncogenic MYC shape tumour-specific gene expression profiles. *Nature* **511**, 483–487 (2014).

34. J. Y. Chen, *et al.*, Hoxb5 marks long-term haematopoietic stem cells and reveals a homogenous perivascular niche. *Nature* **530**, 223–227 (2016).

35. J. Choi, *et al.*, Haemopedia RNA-seq: a database of gene expression during haematopoiesis in mice and humans. *Nucleic Acids Res.* **47**, D780–D785 (2019).
